# The Physiological and Biochemical Response of Ribbed Mussels to Rising Temperatures: Benefits of Salt Marsh Cordgrass

**DOI:** 10.1093/iob/obae031

**Published:** 2024-08-21

**Authors:** A Smith, J Erber, A Watson, C Johnson, W E Gato, S B George

**Affiliations:** B iology Department, Georgia Southern University, Statesboro, GA 30460, USA; Department of Chemistry and Biochemistry, Georgia Southern University, Statesboro, GA 30460, USA; B iology Department, Georgia Southern University, Statesboro, GA 30460, USA; B iology Department, Georgia Southern University, Statesboro, GA 30460, USA; Department of Chemistry and Biochemistry, Georgia Southern University, Statesboro, GA 30460, USA; B iology Department, Georgia Southern University, Statesboro, GA 30460, USA

## Abstract

Salt marsh ecosystems are heavily reliant on ribbed mussel (*Geukensia demissa*) populations to aid in rapid recovery from droughts. The focus of this study was thus to document the effects of rising temperatures on ribbed mussel populations in a Georgia salt marsh. Seven lab and eight field experiments were used to assess the effects of current air temperatures on mussels at two high marsh (HM) sites with short and sparse cordgrass and one mid marsh (MM) site with tall and dense cordgrass. Field results in 2018 and 2019 indicate that ribbed mussels were experiencing extremely high temperatures for prolonged periods of time at the landlocked high marsh (LHM) site. In 2018, the highest temperature (54°C) and longest high temperature events, HTEs (58 days), that is, consecutive days with temperatures ≥40°C, were recorded at this site. When laboratory temperatures were increased from 20 to 36°C, mean heart rates increased by an average of 19 bpm for mussels from both high and MM sites respectively. When field temperatures rose from 20°C in April to 40°C in September 2019, mean heart rates increased by an average of 10 bpm for HM mussels and by 26.3 bpm for MM mussels. Under identical laboratory and field conditions, mean heart rates for mussels from the LHM site with the highest temperatures, increased by <1 bpm and 3.7 bpm respectively. Evidence of the potential role of shade on mussel aggregates was provided by examining whether mussels from the edge of mussel aggregates with little to no cordgrass for shade were more stressed than those living at the center of mussel aggregates. In the absence of shade, mean body temperatures for mussels at the edge of mussel aggregates were up to 8°C higher than for those living in the center underneath a dense tuft of cordgrass. Despite high body temperatures, mean heart rates and Hsp70 gene expression were lower for mussels living at the edges. This agrees with the strategy that during prolong exposure to high temperatures, mussels may reduce their heart rate to conserve energy and enhance survival. Alternatively, heat-stressed mussels at the edges of aggregates may not have the resources to express high levels of Hsp70. Increase in the frequency, intensity, and duration of HTEs may stress the physiological and biochemical function of mussel populations to the limit, dictate mussel aggregate size, and threaten the functionality of SE salt marshes.

## French Abstract

Les écosystèmes des marais salés dépendent fortement des populations de moules côtelées (*Geukensia demissa*) pour aider à se remettre rapidement des sécheresses. L'objectif de cette étude était donc de documenter les effets de la hausse des températures sur les populations de moules côtelées dans un marais salant de Géorgie. Sept expériences en laboratoire et huit expériences sur le terrain ont été utilisées pour évaluer les effets des températures actuelles de l'air sur les moules dans deux sites de haut marais avec de la spartine courte et clairsemée et un site de milieu marais avec de la spartine haute et dense. Les résultats obtenus sur le terrain en 2018 et en 2019 indiquent que les moules côtelées ont connu des températures extrêmement élevées pendant de longues périodes sur le site enclavé du haut marais. En 2018, les événements de température la plus élevée (54°C) et les plus longs événements de haute température, les HTE (58 jours), c'est-à-dire des jours consécutifs avec des températures de ≥40°C, ont été enregistrés sur ce site. Lorsque les températures en laboratoire sont passées de 20 à 36°C, la fréquence cardiaque moyenne a augmenté en moyenne de 19 battements/min pour les moules des sites de haut et moyen marais, respectivement. Lorsque les températures sur le terrain sont passées de 20°C en avril à 40°C en septembre 2019, la fréquence cardiaque moyenne a augmenté en moyenne de 10 bpm pour les moules des marais hauts et de 26,3 bpm pour les moules des marais moyens. Dans des conditions identiques en laboratoire et sur le terrain, la fréquence cardiaque moyenne des moules du haut marais enclavé où les températures ont été les plus élevées a augmenté de <1 bpm et de 3,7 bpm respectivement. La preuve du rôle potentiel de l'ombre sur les agrégats de moules a été fournie en examinant si les moules de bordure des agrégats de moules avec peu ou pas de spartine pour l'ombre étaient plus stressées que celles vivant au centre des agrégats de moules. En l'absence d“ombre, la température corporelle moyenne des moules à la lisière des agrégats de moules était jusqu”à 8°C plus élevée que celle des moules vivant au centre, sous une touffe dense de spartine bovine. Malgré des températures corporelles élevées, la fréquence cardiaque moyenne et l'expression du gène Hsp70 étaient plus faibles chez les moules vivant sur les bords. Cela correspond à la stratégie selon laquelle lors d'une exposition prolongée à des températures élevées, les moules peuvent réduire leur fréquence cardiaque pour conserver leur énergie et améliorer leur survie. Par ailleurs, les moules soumises à un stress thermique en bordure des agrégats peuvent ne pas avoir les ressources nécessaires pour exprimer des concentrations élevées de Hsp70. L'augmentation de la fréquence, de l'intensité et de la durée des HTE peut stresser la fonction physiologique et biochimique des populations de moules jusqu'à la limite, dicter la taille des agrégats de moules et menacer la fonctionnalité des marais salés du sud-est.

## Introduction

Rising temperatures (IPCC report 2023; Matthews and Wynes 2022; McCay et al. 2022; Heeter et al. 2023) and associated mass mortality events pose a significant threat to many organisms (Crotty et al. 2020; Wells et al. 2023). Sessile organisms are specifically at risk of mass mortality events especially those found in the intertidal zone where they are constantly exposed to temperature extremes during the immersion and emersion cycle (Harley et al. 2006; Harley 2008, 2011; Nancollas and Todgham 2022). When extremely high temperatures coincide with emersion in the middle of the day, it can be devastating for sessile organisms such as mussels. For instance, at body temperatures of 30 to 40°C, mortality rates for the blue mussel (*Mytilus edulis*) can exceed 50% within 1 h (Tsuchiya 1983, Seuront et al. 2019).

Unfortunately, marine heatwaves, periods of exceptionally warm temperatures lasting weeks, months, or years (Hobday et al. 2018, 2023) are increasing in frequency (Stillman 2019; Seuront et al. 2019; Hobday et al. 2023). Multiple moderate heatwaves led to the deaths of approximately nine million mussels in the eastern English Channel in the summer of 2018 (Seuront et al. 2019). Similar heatwaves with temperatures reaching 50°C (Heeter et al. 2023; White et al. 2023) led to an estimated loss of over 1 million bay mussels (*Mytilus trossulus*) within a 100-m stretch of shoreline in the Pacific Northwest in 2021 (White et al. 2023).

Microhabitats that provide shade, e.g., crevices in the rocky intertidal and grasses in salt marshes, can mitigate some negative effects of increasing temperatures. For instance, peak temperatures at low tide differ between the surface (>40°C) and interior (<25°C) of mussel aggregates of the California mussel (*Mytilus californianus*) living in the rocky intertidal (Jurgens and Gaylord 2016). They suggested that at low tide those in the interior of aggregates are buffered from solar-driven warming by shading. In salt marsh ecosystems, it has been well documented that the formation of large mussel aggregates is associated with high densities of cordgrass (*Spartina alterniflora*) that provide shade (Bertness 1984; Angelini et al. 2015; Honig et al. 2015; Bilkovic et al. 2017; Derksen-Hooijberg et al. 2017; Hensel et al. 2021).

Several mussel species are known to be quite tolerant of extreme thermal stress. For example, *M. californianus*, can rapidly acquire heat tolerance for up to 3 weeks after being exposed to sublethal temperatures for 2 h at 30, 35, and 40°C (Moyen et al. 2020, 2022). They concluded that this adaptive strategy could be beneficial as temperatures continue to rise. The ability of mussels to acclimate to rising temperatures stems from the upregulation of heat shock proteins, hsps (Snyder et al. 2001). When mussels are exposed to high temperatures, they increase the transcription of hsp genes to produce more hsp (Liu and Chen 2013; Hassan et al. 2019). Heat shock cognates (hscs), that function as molecular chaperones, are also upregulated as temperatures increase during the summer (Hamdoun et al. 2003; Franzellitti and Fabbri 2005; Liu and Chen 2013). For *Mytilus galloprovincialis* when temperatures rise, hsp70 expression increases but hsc70 expression remains unchanged (Franzellitti and Fabbri 2005). They suggest that for long-term protection, some species may express hsc70 genes continuously. Hsp70-3 also plays a crucial role in protein homeostasis, including protein folding, degradation, and disaggregation (Serlidaki et al. 2020). Despite its similarity to other hsp70 isoforms, hsp70-3 may have unique cellular functions and interactions with co-chaperones (Lotz et al. 2019; Serlidaki et al. 2020). It has a role in stabilizing specific mRNAs containing AU-rich elements, which may contribute to its cytoprotective effects during cellular stress (Kishor et al. 2013). Studies of this nature for the salt marsh mussel *Geukensia demissa* are scanty (see Fields et al. 2014, 2016, Fields and Eraso 2020).

*Geukensia demissa* is a keystone species in salt marshes along the east and gulf coast of the United States. They enhance species diversity and ecosystem multifunctionality (Angelini et al. 2015). Densities range from over 2000 individuals/m^2^ in New England salt marshes (Honig et al. 2015) to as low as 99 individuals/m^2^ in mid-Atlantic salt marshes (Kreeger et al. 2015). In South Atlantic salt marshes, densities range from ∼8000 individuals/m^2^ in Virginia (Bilkovic et al. 2017) to ∼200 individuals/m^2^ in Georgia (Williams et al. 2023). Their high densities enhance substrate stability and decrease pollution through the filtration of over 92 metric tons of suspended matter per hectare per year at some sites (Kreeger et al. 2015; Angelini et al. 2016; Crotty and Angelini 2020). They can tolerate an extensive range of temperatures (−22 to 56°C) from Canada to South America (Lent 1968, 1969). However, at body temperatures of 45°C, Jost and Helmuth (2007) noted a dramatic increase in mortality, and a few moments at body temperatures of 50°C can lead to 100% mortality. Models indicate that in the absence of *G. demissa*, it could take up to 100 years for southeastern salt marshes to recover from drought (Angelini et al. 2015, 2016) or in some cases, the salt marsh may never recover (Hensel et al. 2021). To our knowledge, studies that specifically examined how increases in environmental temperatures within and among salt marsh locations affect the physiological and biochemical response of *G. demissa* are scanty.

The focus of this study was thus to address this knowledge gap by documenting the current environmental temperatures at various locations in a southeastern salt marsh and accessing whether the ribbed mussel *G. demissa* living in these locations are experiencing and responding similarly to rising environmental temperatures. Seven lab and eight field experiments were used to assess the effects of current temperatures on mussels living in (a) two HM sites with short and sparse cordgrass and small mussel aggregates and one mid marsh (MM) site with tall and dense cordgrass and large mussel aggregates, (b) eight mussel aggregates located along a transect line at varying distances from a nearby creek in the MM, and (c) the centers of these eight MM mussel aggregates with tall and dense cordgrass and their edges with very little cordgrass. Based on studies by Helmuth (1998) that mussels in large aggregates experience lower body temperatures and tall and dense cordgrass provide shade (Bilkovic et al. 2017; Bilkovic et al. 2021; Hensel et al. 2021), we hypothesized that the landlocked high marsh (LHM) site and the edges of mussel aggregates will have the highest temperatures and be the most stressful environments, while the centers of mussel aggregates will have the lowest temperatures and be the least stressful. MM mussels living in areas with tall and dense cordgrass especially those living at the centers of mussel aggregates will be the most stressed when exposed to high temperatures in the laboratory and field. Signs of stress will include higher body temperatures, heart rates, hsc70, hsp70, and hsp70-3 gene expression. We also expected that these differences will become more pronounced during the summer months. Through these experiments, we hope to reinforce the threat of rising temperatures on southeastern salt marsh mussels.

## Methods

### Part I: Variation among sites in the high and MM

#### Description of the three sites

**Fig. 1:**
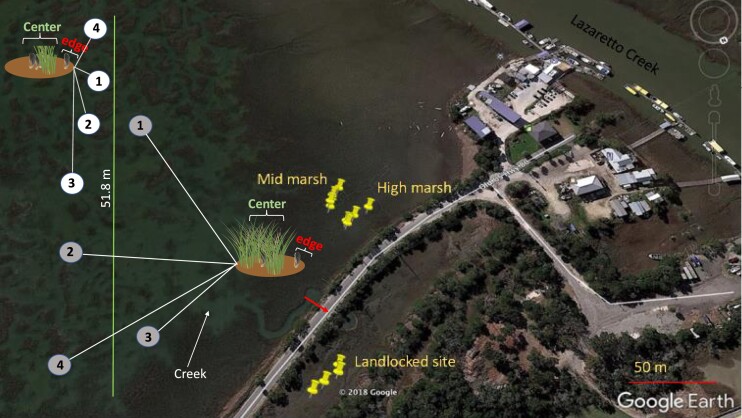
Map of Tybee Island, Georgia showing the three sites. Old US Hwy 80 referred to as Old Tybee Road at Tybee Island, GA, divides the first two sites used in the study into two, with the landlocked high marsh (LHM) site on the right and the high marsh site (HM) on the left side of the road. The third site, in the mid marsh (MM) is on the same side as HM. There were four plots at each site. The short arrow shows the connection (via a culvert under the road) between the landlocked high marsh site and the main marsh (HM and MM). A 51.8 meter transect line was placed approximately 30 m from Old Tybee Road. The white (exposed aggregates) and grey (shaded aggregates) circles are the approximate locations of 8 mussel aggregates in the mid marsh used in Part II a and b (methods). Distance between aggregates varied from 2 m to 13 m. Schematic diagrams of the center and edges of aggregates on shaded and exposed aggregates are shown.

Ribbed mussels (*G. demissa*) were collected from three sites at Tybee Island, Georgia (32.000517 N, −80.845767 W) in 2018 and 2019 for five laboratory and three field experiments (Table 1). Site 1 is completely enclosed and sandwiched between two major roads (hereafter referred to as landlocked high marsh site or LHM, Fig. 1, Table 2; Supplementary Fig. S1). A culvert placed under one of the roads connects the main HM zone to site 1 (Fig. 1). This site is characterized by very short and sparse cordgrass (*S. alterniflora*) and few mussel aggregates (Table 2). Site 2 is in the main HM located across the road from site 1, with short but slightly more cordgrass and larger mussel aggregates than at site 1. Site 3 is in the mid marsh (MM) furthest away from site 1 (>100 m away), with the tallest and highest % cover of cordgrass and the largest mussel aggregates (Fig. 1, Table 2).

**Table 1:** Details of seven laboratory and eight field experiments to document current environmental temperatures and assess possible effects on mussels living in various locations within a southeastern US salt marsh

Type and (number) of experiments	Temperatures used	Number of mussels/site/month	Total number of mussels*, aggregates, locations	Collection dates or field work
Part I: Heart rate measurements at three sites (high, mid, and landlocked high marsh zones)				
Lab (3)	20, 30, 36°C	10/site, 30/month	90	October and November 2018, April 2019
Field (3)	20, 20, ≥40°C	10/site, 30/month	90*	October 2018, April and September 2019**
Part IIa: Heart rate measurements from eight mussel aggregates in the mid salt marsh				
Lab (1)	36°C	5/aggregate***	4 exposed/4 shaded	August 2019
Lab (1)	36°C	5/aggregate***	4 exposed/4 shaded	September 2019
Part IIb: Mussel body temperature at the edge and center of eight aggregates in the MM				
Field (5)	NA	20/aggregate****	10 from the center, 10 from the edge of each aggregate	Mar, May, July, and Aug 2019, May 2021****
Part IIc: Hsc70/hsp70/hsp-3 gene expression for mussels from the edge and center of an aggregate				
Lab (2)	20, 36°C	14	7 from the center, 7 edge	May and July 2021

* Total number of mussels used in field expt. decreased in some cases due to no signal detected.

** In September, mussels were not exposed to laboratory conditions, heart rates were measured in the field.

*** A total of 20 mussels from exposed aggregates and 20 from shaded aggregates for a total of 40/month.

**** A total of 20 mussels/aggregate, 160 mussels/month + 40 mussels in May 2021 (total = 680).

**Table 2:** Salinity, mean height and % cover of the cordgrass *Spartina alterniflora*, and mussel aggregates at three locations (LHM, HM and MM) off old Tybee Road, Tybee Island, Georgia. There were 4 plots per location, and 10 cordgrass stems were measured in each plot for a total of 40 measurements/site. Values are mean ± standard deviation

Salt marsh sites	Number of plots (n)	Salinity (‰)	Mean cordgrass height (cm)	Mean cordgrass coverage (%)	Mussels/aggregate
Land-locked high marsh (LHM)*	4	33	38.8 ± 5.9	45.0 ± 8.1	1–15
High marsh (HM)**	4	33	44.4 ± 9.4	48.0 ± 11.9	15–50
Mid marsh (MM)***	4	34	61.0 ± 2.0	84.0 ± 2.5	100–300

* Wet, high percentage of coarse sand, occasionally with standing water at low tide.

** Wet, muddy, with coarse sand.

*** Wetter, muddier, low percentage of coarse sand.

#### Field work, mussel collection, and lab maintenance

In the field, four 1 × 1 m plots were randomly selected at each of the three sites (Fig. 1). Each plot had a mussel aggregate (i.e., raised portions of the salt marsh substrate, composed of small to large numbers of individuals at the base of cordgrass stems). Temperature data were recorded at 15-min intervals with HOBO pendant MX Temperature/Light Data Loggers and HOBO TidbiT MX 2203 Temperature 400’ Data Loggers mounted on PVC pipes, placed 46 cm above the salt marsh sediment in close proximity to mussel aggregates at each site (five in the MM, one in the HM and one in the LHM site in 2018 and, six in the MM, two in the HM, and two in the LHM site in 2019). The data loggers were placed at a height on the PVC pipes that ensured that they were close to mussels on the sediment surface and covered during high tide. In the southeastern United States, there are generally two low and two high tides. Loggers were thus submerged at high tide and exposed at low tide, providing air and water temperatures. During each field trip, data from each logger were downloaded using the HOBO app on a Samsung Note 4. Dataloggers that malfunctioned or lost were replaced on subsequent trips. All field trips for this study were made at low tide.

To measure the heart rate of mussels, two to three mussels that measured between 8.9 and 11.6 cm in length were collected from each aggregate for a total of 10 mussels per site (30 per month) in October and November 2018, and April and September 2019 (Tables 1 and 2).

Before heart rate measurements, mussels were kept submerged in three containers (45 × 30 × 30 cm) for 10–16 days (10 mussels/container/site) at room temperature (20°C). Each tank was aerated, temperature measured with a handheld thermometer, and salinity with a handheld refractometer. Salinity was kept between 30 and 33‰, matching salt marsh salinities. The mussels were initially placed in containers with salt marsh sediment and unfiltered sea water containing algae to feed. To ensure high mussel survival in the laboratory, they were transitioned to a high-quality algal shellfish diet (Shellfish Diet 1800, Reed Mariculture, Campbell, CA, USA) used in many shellfish studies (Braby and Somero 2006; Moyen et al. 2020). They were fed three times a week, based on the manufacturer's instructions, and tanks cleaned twice a week. Survival in the lab was 100%.

#### Method used to measure heartbeat of mussels

Heart rate was measured using a non-invasive technique developed by Depledge et al. (1996) and modified by Burnett et al. (2013). An infrared emitter and phototransistor sensor was placed on each mussel's shell, just above the heart located near the mid-dorsal posterior hinge, and cardiac activity recorded for 60–80 s (Supplementary Fig. S2). The signal was amplified using AMP-03 (Newshift LDA, Leiria, Portugal) and recorded with a Picoscope 2207B data acquisition system (Picotechnology, Cambridge, UK). Heart rates were calculated by manually counting the data wave forms in Picoscope software (v.6.10.6.2) and expressed as beats per minute (bpm).

#### Heart rate measurements in the laboratory

To test whether ribbed mussels from the three sites will respond similarly to an acute heating event, mussel heart rate was measured in a series of laboratory experiments; the first set of experiments in October 2018. Two water temperature treatments were used: 20°C, 36°C (Table 1). These temperatures were selected based on temperatures observed at the three sites. For mussels at 20°C, the water in each tank was siphoned out and ribbed mussel heart rate recorded by placing the sensor just above the heart. For the 36°C treatment, mussels were transferred to a water bath (30 × 15 × 15 cm). To keep mussels from each site separate, the water bath was separated into three levels by Rubbermaid sink mats that were cut and fit to make three levels (10 mussels/level/site). Ten mussels from the LHM site were placed on the lowest level of the water bath, 10 from the HM site on the middle level, and 10 from the MM site on the topmost level. They were placed in an orientation that allowed the heartbeat to be recorded with very little disturbance. Over the course of 1 h, the water temperature increased gradually from 20 to 36°C and maintained at 36°C for 30 min. This ramping rate (>10–16°C/hr ) was higher than the rate at which temperatures increase in the field during emersion and immersion at Tybee Island and considered acute. After 1 h and 30 min, the water level was lowered with a siphon to expose the first 10 mussels to the air. The sensor was then placed just above the heart and heart rate measured for 1 min for each individual. This was repeated for mussels from the LHM and HM sites in the lower levels. Duplicate measurements were made for each mussel. Measurements for all mussels were recorded within 45 min. This experiment was repeated in November 2018 at temperatures of 20 and 36°C and in April 2019 at temperatures of 20 and 30°C. There were no laboratory experiments in September 2019 (Table 1).

#### Heart rate measurements in the field

After each laboratory experiment, mussels were returned to their original field site for *in situ* measurements of heart rates. Individuals were placed upright in the substrate, leaving up to 3 cm of the top of the mussel visible to allow for placement of the heart rate sensor. To account for transportation and handling effects, mussels were allowed to recover for 30 min to 1 h before heart rate measurements were made. The Picoscope, amplifier, and sensors were housed in a waterproof container, and hooked up to a Macintosh laptop when ready for use. Heart rates were measured as described above, recording data from the HM first, MM second, and the LHM site third. The heart rate of mussels from the LHM site were measured last because this site remained submerged for a longer period than the other two sites even at the lowest tides. Duplicate measurements were made for each mussel. Each month, one observer was responsible for securing the sensor on the mussels and a second observer was responsible for all recordings using Picoscope software on a Macintosh laptop. Due to weather conditions in November 2018, it was impossible to carry out field experiments. Field experiments were thus carried out in October 2018 (20°C), April 2019 (20°C), and September 2019 (≥40°C). In October 2018, no heart signal was detected for one mussel in the MM; in April 2019, six (two mussels/site), and in September 2019, no heart signal was detected for a total of 18 mussels (two mussels in the LHM, eight in the HM, and eight in the MM).

#### Data analysis

To determine which of the three sites was the most stressful environment, temperature logger data collected every 15 min were used to estimate the consecutive number of days when temperatures were  ≥40°C for each site. This was hereafter referred to as high temperature events (HTEs). To determine whether air temperatures differed significantly among sites, temperature logger data were extracted between 12:00 PM and 3:15 PM when temperatures were rising and reached their peak in August and September 2019. The data were analyzed with a two-way analysis of variance (ANOVA) with interaction. Sites (3) and date (August 30, August 31, and September 17) were fixed factors.

To ascertain whether heart rates of mussels from the MM were higher when exposed to laboratory temperatures of 30 or 36°C and field temperatures >40°C, laboratory data for October and November and field data from October and April were analyzed separately then combined. Combined laboratory data were analyzed with a 3 × 2 × 2 design (site: HM, MM, LHM; month: October, November; experiment type: lab 20°C and lab 36°C). Separate laboratory and field comparisons were made for each month with a 3 × 2 design (site: HM, MM, LHM; experiment type: lab, field). Laboratory and field data were then combined and analyzed with a 2 × 3 × 2 design (Month: October, April; site: HM, MM, LHM; experiment type: lab, field). For all designs, repeated measures ANOVA were used with experiment type, site, and month as fixed factors, mussels as a random factor, with their interactions. The afex and sdamr packages in R were used in the analysis. Mauchley's test was carried out and Greenhouse–Geisser (df_gg_) correction applied to the degrees of freedom to correct for problems of nonsphericity (Singmann et al. 2023). Effect sizes were reported as generalized eta squared (ges). Mussel mortality was zero during all laboratory experiments leading to completely balanced designs.

Data from three months of field work were combined and analyzed with a 3 × 3 (month: October 2018, April 2019, September 2019; site: HM, MM, LHM) mixed model ANOVA design (type 3 test) with interaction between site and month. To control for type 1 errors, the denominator degrees of freedom were calculated using the Satterthwaite's and the Kenward–Roger approximations. For all analyzes, when interactions were significant, they were interpreted instead of the main effects. Post-hoc comparisons were made with the emmeans and multcompview packages in R and Rstudio version 2024.04.0 + 735 (Singmann and Kellen 2019; Meier 2022; Speekenbrink 2023). Data were initially screened for equality of variances (O'Brien and Levene test) and normality (Shapiro–Wilk test).

### Part II: Variation among eight mussel aggregates in the MM

#### Description of MM mussel aggregates

To test whether a mussel's response to high temperatures was affected by its location in the MM or on a mussel aggregate, eight mussel aggregates were selected along a 51.8-m transect line at ∼30 m from Old Tybee Road (Fig. 1, Table 1). Aggregates located close to a nearby creek were larger and in areas with tall and dense cordgrass, while those located further from the creek were smaller and in areas with short and sparse cordgrass (Table 3). Five temperature loggers were placed close to mussel aggregates (see Part I. section b).

**Table 3:** Characteristics of eight salt marsh aggregates in the MM zone, at Tybee Island, Georgia.

Aggregate exposure	Aggregate	Aggregate	Distance away from	Sediment characteristics and	*Cordgrass*	Mussels
Type	replicate	Size*	Creek along transect	Cordgrass coverage	height (cm)**	numbers***
Exposed	1	Small	∼50 m	Driest and sparse cordgrass	62.3 ± 2.1	101 ± 46
Exposed	2	Small	∼32 m	Drier and sparse cordgrass	59.1 ± 2.1	68 ± 12
Exposed	3	Small	∼26 m	Drier and sparse cordgrass	62.4 ± 2.1	117 ± 23
Exposed	4	Small	∼17 m	Wet, muddy, less dense cordgrass	59.6 ± 2.1	86 ± 15
Shaded	1	Large	∼8 m	Wet, muddy, tall and dense cordgrass	75.6 ± 2.1	144 ± 30
Shaded	2	Small	∼26 m	Wet, muddy, tall and dense cordgrass	66.3 ± 2.1	125 ± 35
Shaded	3	Large	∼7 m	Wet, muddy, tall and dense cordgrass	72.3 ± 2.1	124 ± 20
Shaded	4	Large	<5 m	Wet, muddy, tall and dense cordgrass	69.6 ± 2.1	123 ± 37

* Aggregate area: Small 1.5–3.5 m^2^, large 4–5.0 m^2^.

** Values are means ± standard error, *n* = 240 (10 stems/month/aggregate).

*** Values are means ± standard deviation, *n* = 32 (4 months/aggregate).

#### Field collection and laboratory maintenance

To determine whether the heart rate of mussels differed with location in the MM, five mussels were collected from eight aggregates located along the transect line (four large aggregates with tall and dense cordgrass, shaded (*n* = 20), and four aggregates with short and sparse cordgrass, exposed (*n* = 20; Fig. 1, Table 3). All mussels were similar in size (10.0 ± 0.6 cm in length, *n* = 40 in August 2019; 10.1 ± 0.6 cm in length, *n* = 40 in September 2019, respectively).

#### Heart rate measurements in the laboratory

In the laboratory, mussels from the eight aggregates were placed in eight plastic tanks (five mussels/tank) containing approximately 3 L of water. The tanks were aerated and salinity (35–38‰) matched field salinities at the time of collection and kept constant throughout the lab experiments. The mussels were fed 2.8 mL per tank of Shellfish Diet 1800™ three times per week, and tanks cleaned twice per week. Laboratory temperatures were between 18 and 22°C.

To measure the heart rate of mussels, we created two levels in a water bath with Rubbermaid mats (see Part 1 section c and d). Each level was set up with 10 mussels (five mussels from an exposed aggregate and five from a shaded aggregate). The mussels were placed into the water bath at a starting temperature of 22°C. The water bath temperature was gradually increased from 22 to 36°C in 1 h (see part 1, section c and d). As in Part I section d, the water temperature was maintained at 36°C for 30 min. After 30 min, the water level in the water bath was lowered to reveal the top layer of mussels for heart rate measurements. The procedure was repeated for mussels in the lower level. Mussel heart rates were recorded in August and September 2019 (Table 1).

#### Mussel body temperature measurements from the edge and center of mussel aggregates

To determine whether mussels from the edges of a mussel aggregate with little to no cordgrass would have higher body temperatures than those from the center with high cordgrass density, the temperatures of 20 mussels/aggregate (10 mussels from the edge and 10 from the center of each of the 8 aggregates; see Fig. 1 and part II, section a) were measured with a non-invasive FLUKE 62 MAX infrared temperature gun. The distance between the center and edge of each aggregate was generally <0.5 m. A total of 160 measurements were made per month in March, May, July, and August 2019 and an additional 40 measurements in May 2021 for a total of 680 mussels (Table 1). Every month, body temperature measurements were made during the same time of day. The pandemic prevented field work in 2020 and limited the amount of field work that could be done in 2021.

#### Heat shock gene expression of mussel gills from the edge and center of a mussel aggregate

To determine whether hsp70, hsp70-3, and hsc70 gene expression of mussels from the center and edge of an aggregate differed, six mussels (three from the edge and three from the center) were collected in May 2021 and eight (four from the edge and four from the center) in July 2021 (Table 1). In the laboratory, mussels were separated into two groups: controls (20°C) or subjected to a temperature of 36°C in a water bath for 1 h and a half following procedures used in Part II section b. All of the mussels were then sacrificed, and their gill tissues stored at −80°C until ready for analysis.

For analysis of gene expression, total RNA isolation was completed using the RNeasy Mini by Qiagen. Approximately 20–30 mg of each gill tissue sample was added to 900 µL of QIAzol lysis reagent for homogenization and bonded to the RNA spin column. The purity, via 260/280 ratio, and concentration of the total RNA of each sample was examined with a Nanodrop (Thermo Fisher Scientific Nanodrop, 2000/2000c Spectrophotometer) nucleic acid spectrophotometer. In addition, the quality of the total RNA was determined using RNA gel electrophoresis stained with ethidium bromide.

The expression of hsp70, hsp70-3, and hsc70 genes for mussels from the aggregate center and edge was determined in duplicate and repeated three times, using quantitative real-time quantitative polymerase chain reaction. The selected genes and their associated accession numbers were obtained from prior studies on heat shock genes for the Mediterranean mussel, *M. galloprovincialis* (Kourtidis et al. 2006, Table 4; Franzellitti et al. 2020), as no prior hsp70 sequences were available for the ribbed mussel *G. demissa*. The forward and reverse primers were purchased from Integrated DNA Technologies (IDT) Inc. Bio-Rad iScript Reverse Transcription Supermix for RT-qPCR was used to synthesize cDNAs from total RNA. Primers and SsoFast EvaGreen Supermix were combined with the cDNAs for the RT-qPCR. Bio-Rad CFX^96^ system was used to assess the relative gene expression according to the manufacturer's guidelines. Each run included no template control to account for false positives.

**Table 4:** Accession number and forward and reverse primer sequences for selected heat shock genes (Kourtidis et al. 2006; Franzellitti et al. 2020).

Gene	Accession #	Forward primer	Reverse primer
HSP70	AY861684	CTGCTTGTGAAAGGGCAAAG	CTCTTGGTGCTGGAGGTATTC
HSP70-3	AJ783712	GCTCCTTTGTCCCTTGGTATT	GAGTCTTCCTCTGTCATTGGTG
HSC70	AJ783715	CTGCTTGTGAAAGGGCAAAG	GTGGAAACCGCGAATGAATG

#### Data analysis

To determine whether the heart rate of mussels was influenced by the location of the aggregate along a 51.8-m transect line in the MM, a three-way ANOVA with interaction was used. Aggregate exposure (exposed, shaded), aggregate replicate along the transect line (four), and month (August, September) were main factors.

To ascertain whether mussels from the aggregate edge had higher body temperatures than those from the aggregate center, data were analyzed with a two-way analysis of variance (ANOVA) for unequal sample sizes with aggregate replicate (eight) and mussel location on an aggregate (center, edge) as fixed factors, including their interaction.

Three-way ANOVAs without interaction were used to analyze mean relative quantity for each hsp and hsc gene from the gill tissues of mussels collected from the center and edges of a mussel aggregate. Month (May, July), location on aggregate (aggregate center, aggregate edge), and laboratory treatment (controls 20°C, 36°C) were fixed factors. When differences were statistically significant, pairwise comparison of means were made using the Tukey HSD test (Sokal and Rohlf 2014). All data were tested for equality of variances (O'Brien and Levene Tests), normality (Shapiro–Wilk test), and the absence of two- and three-factor interactions before running the 3-Way ANOVA without interaction (Neter et al. 1990). Data were analyzed using JMP Pro 17 and figures prepared using R software 4.3.2. 2023 and Rstudio version 2024.04.0 + 735.

## Results

### Part I: Variation among the three sites

#### Frequency and duration of HTEs

**Fig. 2:**
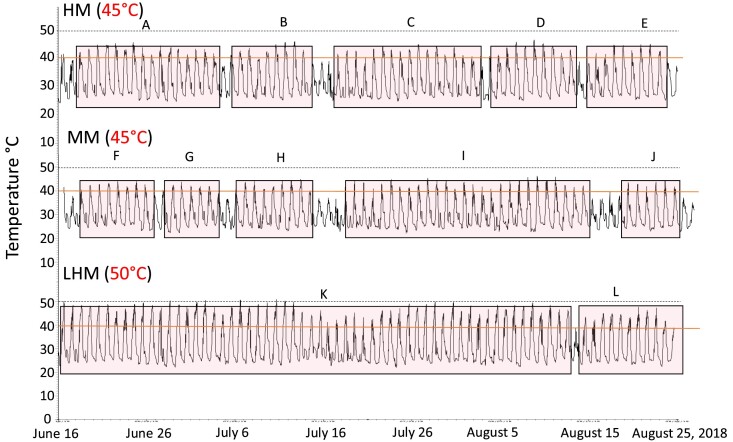
Temperature measurements summer 2018. Hobo data loggers were placed in the High Marsh (HM), Mid Marsh (MM) and Landlocked High Marsh (LHM) sites at Tybee Island, Georgia, from June through August 2018; Data were collected at 15-minute intervals. The number of days when temperatures were at or above the 40°C line constitutes a high temperature event (HTE); that is, the number of days when air temperatures were ≥ 40°C separated by a day or more of air temperatures < 40°C at the three sites. Temperatures reached the 50°C dashed line at the LHM site. Highlighted boxes (A–L) are distinct HTEs at each site. The highest temperature recorded at a site are in parentheses.

**Fig. 3:**
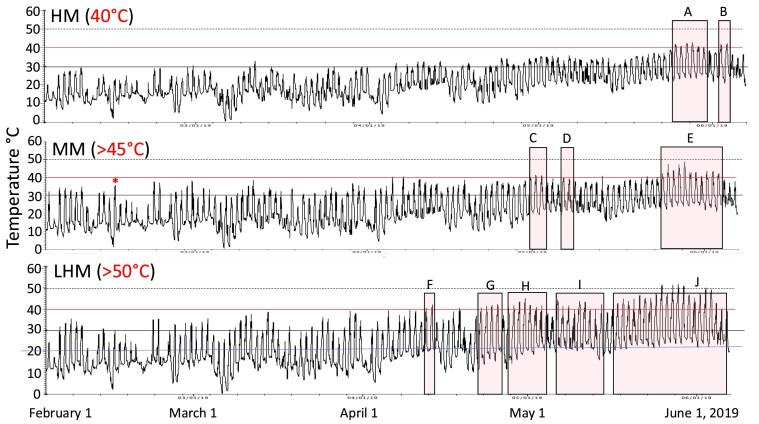
Temperature measurements winter and spring 2019. Hobo data loggers were placed in the HM, MM and LHM sites from February through early June 2019. The asterisk represents the largest difference in daily maximum and minimum temperatures observed at the MM site. Temperatures above the 30°C line were rare until April at the HM site. See Fig. 2 for more details.

**Fig. 4:**
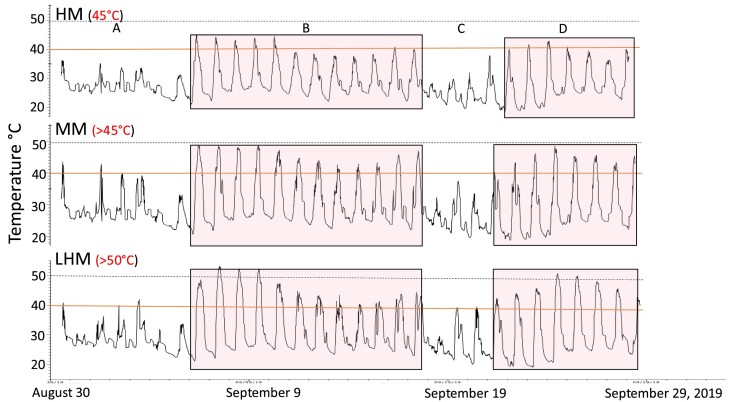
Temperature measurements summer 2019. Hobo data loggers were placed in the HM, MM and LHM sites at Tybee Island, Georgia, in August and September 2019. See Fig. 2 for more details.

The magnitude, duration, and temporal variation of air temperatures from loggers placed close to mussel aggregates differed among sites. The number and duration of HTEs during summer 2018 (June –August), winter and spring 2019 (April –June), and summer 2019 (August–September) was always lowest at the HM and highest at the LHM site (Figs. 2–4, respectively). For instance, during the summer months of 2018 (June–August), five distinct HTEs, were recorded in the high and MM and two in the LHM site (Fig. 2A–L). The longest sequence of HTEs was 16 days in the HM, 27 days in the MM, and 58 days at the LHM sites (Fig. 2A, I, and K).

In 2018, temperatures were  ≥45°C for 4 days in the MM, 6 days in the HM, and 48 days in the LHM site (Fig. 2). Temperatures ≥50°C were only recorded at the LHM site; with five days with temperatures ≥50°C in June and July 2018, late May, early June 2019, and September 2019, respectively (Figs. 2–4). In both years, the maximum air temperature recorded at the LHM site was 54°C.

Air temperatures above 30°C during the winter months of 2019 (February–early March) were rare in the HM but common in the MM and LHM sites (Fig. 3). Daily swings in temperature of 20°C (high marsh) to 30°C (MM and LHM) were observed during the daily emersion–immersion cycle. The magnitude of these daily fluctuations was highest in the MM site during the winter when minimum temperatures approached 0°C and maximum temperatures were ∼37°C (Fig. 3).

**Fig. 5:**
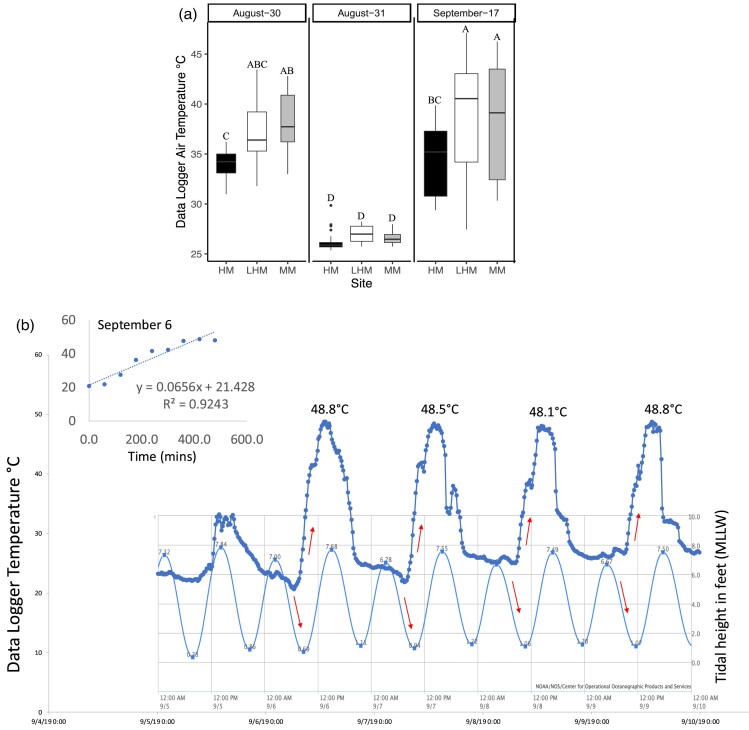
(a and b) Boxplots of air temperatures and tidal height in feet in September 2019. (a) Box plots of air temperature data between 12:00 PM and 3:15 PM extracted from temperature data loggers placed in the HM, MM, and LHM sites in August and September of 2019. The length of the box is the interquartile range; the bottom and top of the box are the 25th and 75th quartiles, respectively; the upper and lower whiskers extend to the maximum and minimum values. When visible the line inside the box is the median. Outliers outside the box are represented by closed circles. Different letters indicate significant differences between sites. (b) Temperatures from data loggers and tidal height in feet for September 5–10, 2019 at the LHM site, Tybee Island, Georgia. Arrows indicate rising temperatures and approaching low tides. Temperature values at the top of the figure are the maximum temperatures recorded during low tide. The insert is a regression of temperature versus time. Tidal height data from NOAA. https://tidesandcurrents.noaa.gov/noaatidepredictions.html?id=TEC3399.

Mean air temperatures from temperature logger data extracted between 12:00 PM and 3:15 PM indicated significant interaction between month and site (*F* = 3.0, *P* = 0.0205; Fig. 5a). On August 30 and September 17, air temperatures were significantly lower at the HM site than at the LHM and MM sites. On August 31, air temperatures did not differ among the three sites (Fig. 5a). A snapshot of the interaction between the tidal cycle and air temperatures between September 5 and 10, 2019 shows that air temperatures were increasing very rapidly as low tide approached between 1:00 PM and 2:00 PM at the LHM site (Fig. 5b). Between September 6 and 8, ramping rate varied between 4 and 5°C/hr.

#### Mussel heart rates: Laboratory comparisons at 20 and 36°C in October and November 2018

**Fig. 6:**
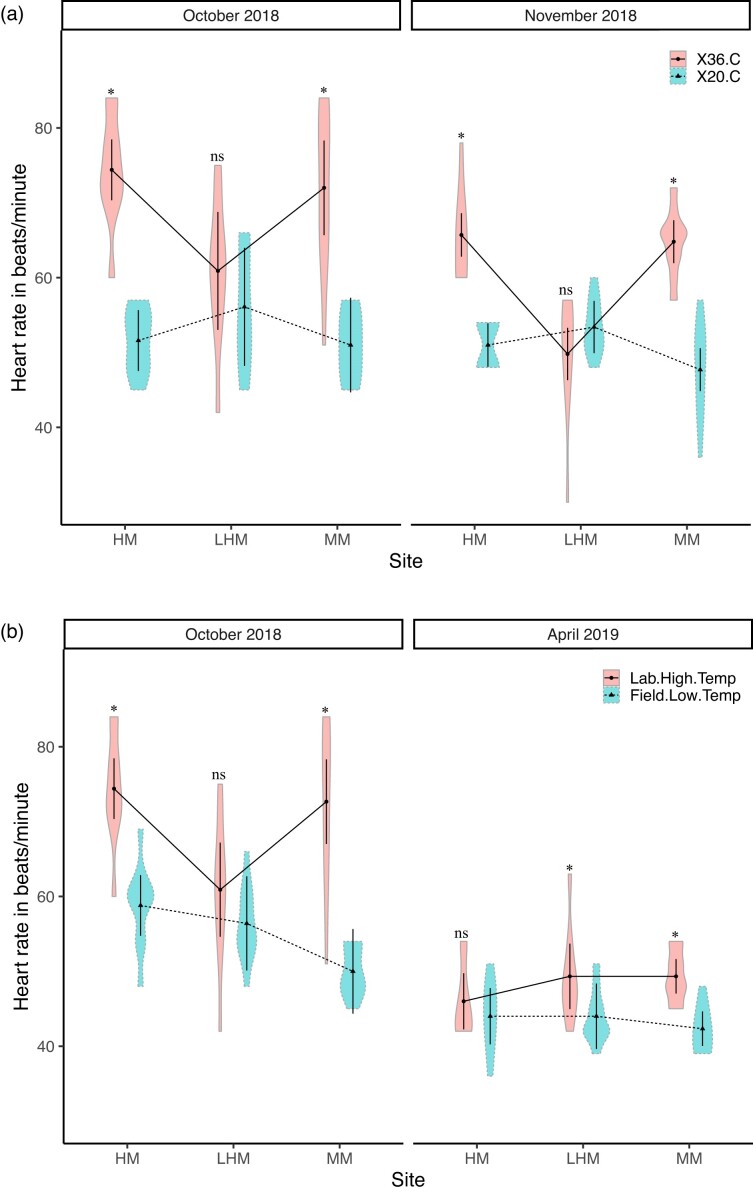
(a and b) Violin plots of heart rate in bpm for the mussel *G. demissa* (a) Laboratory measurements: Violin plots depicting the estimated density of heart rate in bpm that fall within a given interval for mussels from the HM, LHM, and MM sites off of old Tybee Road, Tybee Island, Georgia in October and November 2018. Ten mussels were collected per site for a total of 30 mussels subjected to water temperatures of 20°C and 36°C. Within the violin plots are the within-subject error bars and the estimated marginal means. Solid and dash lines highlight the interaction between site and laboratory temperatures for both months. Asterisks indicate significant differences between temperatures at each site. (b) Laboratory and field measurements: Violin plots of heart rate in bpm for mussels at the LHM, MM, and HM sites in October 2018 and April 2019. Solid and dash lines represent the interaction between site and lab temperatures for October and no interaction in April.

In both months, mussel heart rates did not differ significantly among sites at laboratory temperatures of 20°C (Fig. 6a). The interaction terms (site x lab temperature: *F*_2,54_ = 23.3, *P* < 0.0001 and month x lab temperature: *F*_1,54_ = 7.2, *P* = 0.01) were significant (Table 5a, Fig. 6a). In both months, mussel heart rates increased significantly at 36°C for two of the three sites (Oct: HM, increase of 22.8 bpm, *t* = −7.4, *P* < 0.0001, *df* = 54, MM, increase of 21.0 bpm, *t* = −6.8, *P* < 0.0001 *df* = 54; Nov: HM: 14.7 bpm, t = −4.7, *P* = 0.0002, *df* = 54; MM: 17.1 bpm, *t* = −5.5, *P* < 0.0001, *df* = 54, Table 5b, Fig. 6a). Under identical laboratory conditions, the heart rates of mussels from the LHM site did not increase significantly (Oct: increased by 4.8 bpm, *t* = −1.5, *P* = 0.6341, *df* = 54; Nov: decreased by 3.6 bpm, 1.2, *P* = 0.8524, *df* = 54; Table 5c, Fig. 6a). The highest mean heart rates were recorded for HM and MM mussels (72.0 and 74.0 bpm) and the lowest for LHM mussels (49.8 bpm).

**Table 5:** Three-way repeated measures ANOVA with interaction between site, month, and temperature of the heart rates in bpm for the ribbed mussel *G. demissa* from the HM, MM, and LHM sites at Tybee Island, Georgia in October and November 2018 and subjected to laboratory temperatures of 20 and 36°C. A total of 30 mussels/month, duplicate measurements/mussel for a total 60 measurements/month. Pairwise post-hoc comparisons between site and temperature for each site (b and c) made using the emmeans and multcompview packages in R

a. Overall model					
Effect	df	MSE	F	ges	P value
Month	1, 54	43.32	21.72	0.160	<0.001
Site	2, 54	43.32	7.62	0.118	0.001
Month:Site	2, 54	43.32	0.31	0.005	0.732
Temperature	1, 54	47.95	102.51	0.499	<0.001
Month:Temperature	1, 54	47.95	7.23	0.066	0.010
Site:Temperature	2, 54	47.95	23.29	0.312	<0.001
Month:Site:Temperature	2, 54	47.95	0.33	0.006	0.720
Type 3 tests, Satterthwaite's approximation, ges = generalized eta squared.					
b. October 2018					
Contrast	Difference	SE	df	t.ratio	*P* value
High marsh x 20°C–Landlocked x 20°C	−4.5	2.35	54	−1.912	0.4063
High marsh x 20°C–Mid marsh x 20°C	0.6	2.35	54	0.255	0.9998
**High marsh x 20°C–High marsh x 36°C**	−**22.8**	**3.10**	**54**	−**7.363**	**<0.0001**
High marsh x 20°C–Landlocked x 36°C	−9.3	3.02	54	−3.078	0.0365
High marsh x 20°C–Mid marsh x 36°C	−20.4	3.02	54	−6.753	<0.0001
Landlocked x 20°C–Mid marsh x 20°C	5.1	2.35	54	2.167	0.2701
Landlocked x 20°C—High marsh x 36°C	−18.3	3.02	54	−6.058	<0.0001
**Landlocked x 20°C–Landlocked x 36°C**	−**4.8**	**3.10**	**54**	−**1.550**	**0.6341**
Landlocked x 20°C–Mid marsh x 36°C	−15.9	3.02	54	−5.263	<0.0001
Mid marsh x 20°C–High marsh x 36°C	−23.4	3.02	54	−7.746	<0.0001
Mid marsh x 20°C–Landlocked x 36°C	−9.9	3.02	54	−3.277	0.0215
**Mid marsh x 20°C–Mid marsh x 36°C**	−**21.0**	**3.10**	**54**	−**6.781**	**<0.0001**
High marsh x 36°C–Landlocked x 36°C	13.5	3.57	54	3.786	0.0049
High marsh x 36°C–Mid marsh x 36°C	2.4	3.57	54	0.673	0.9842
Landlocked x 36°C–Mid marsh x 36°C	–11.1	3.57	54	−3.113	0.0333
c. November 2018					
Contrast	Difference	SE	df	t ratio	*P* value
High marsh x 20°C–Landlocked x 20°C	–2.4	2.35	54	–1.020	0.9093
High marsh x 20°C–Mid marsh x 20°C	3.3	2.35	54	1.402	0.7256
**High marsh x 20°C–High marsh x 36°C**	**–14.7**	**3.10**	**54**	**–4.747**	**0.0002**
High marsh x 20°C–Landlocked x 36°C	1.2	3.02	54	0.397	0.9987
High marsh x 20°C–Mid marsh x 36°C	-13.8	3.02	54	–4.568	0.0004
Landlocked x 20°C–Mid marsh x 20°C	5.7	2.35	54	2.422	0.1671
Landlocked x 20°C–High marsh x 36°C	–12.3	3.02	54	–4.071	0.0020
**Landlocked x 20°C–Landlocked x 36°C**	**3.6**	**3.10**	**54**	**1.163**	**0.8524**
Landlocked x 20°C–Mid marsh x 36°C	–11.4	3.02	54	–3.774	0.0051
Mid marsh x 20°C–High marsh x 36°C	–18.0	3.02	54	–5.958	<0.0001
Mid marsh x 20°C–Landlocked x 36°C	–2.1	3.02	54	–0.695	0.9817
**Mid marsh x 20°C–Mid marsh x 36°C**	**–17.1**	**3.10**	**54**	**–5.522**	**<0.0001**
High marsh x 36°C–Landlocked x 36°C	15.9	3.57	54	4.459	0.0006
High marsh x 36°C–Mid marsh x 36°C	0.9	3.57	54	0.252	0.9999
High marsh x 20°C–Landlocked x 20°C	–15.0	3.57	54	–4.207	0.0013

#### Laboratory and field comparisons: October 2018

Mussel heart rates differed significantly in the laboratory and field (*F*_1,24_ = 44.2, *P* < 0.001; Table 6a–d). The interaction between site and experiment type (laboratory and field) was significant (*F* = 6.3, *P* = 0.001). The interaction was mainly due to differences in heart rate response by mussels exposed to high and low temperatures in the laboratory and field (Table 6a–d). At 20°C in the laboratory and field, the heart rates of mussels from the HM site increased significantly in the field (increase of 7.2 bpm, *t* ratio = 3.0, *P* = 0.0169, *df* = 27; Table 6b) but not for those from the MM and LHM sites (increase of 1.2 bpm, *t* = 0.493, *P* = 0.8752; increase of 0.3 bpm, *t* = 0.123, *P* = 0.9917, *df* = 27, respectively, Table 6c and d). At 36°C in the laboratory and 20°C in the field, the heart rates of mussels from the HM and MM decreased significantly (HM: 74.4 ± 2.9 to 58.8 ± 1.5 bpm in the field, *t* =–4.9, *P* = 0.0001, *df*, 27; MM: 72.0 ± 2.9 bpm to 49.8 ± 1.5 bpm in the field, *t* =–6.9, *P* < 0.000, *df* = 27; Table 6b–d, Fig. 6b). Under identical laboratory and field conditions, heart rates did not decrease significantly for mussels from the LHM site (60.9 ± 2.9 to 56.4 ± 1.5 bpm, *t* = −1.4, *P* > 0.3534, *df* = 27; Table 6c, Fig. 6b). Results were identical when data for October and April were combined and analyzed (Supplementary Table S2).

**Table 6:** Two-way repeated measures ANOVA of heart rates in bpm for the ribbed mussel *G. demissa* measured in the laboratory and field at Tybee Island, Georgia in October 2018 and April 2019. Post-hoc comparisons were made between experiment type (laboratory, field) and temperature (high and low) for each site (b–f)

October 2018:					
a. Overall model					
Effect	df_gg_	MSE	F	ges	*P* value
Site	2, 27	37.16	4.10	.073	0.028
Experiment type	1.57, 42.47	67.03	44.16	.547	<0.001
Site:Experiment type	3.15, 42.47	67.03	6.27	.256	0.001
Degrees-of-freedom method: Greenhouse–Geisser					
b. Site = High marsh:					
Contrast	Difference	SE	df	t.ratio	*P* value
Field.20°C–Lab.20°C	7.2	2.43	27	2.958	**0.0169**
Field.20°C–Lab.36°C	−15.6	3.21	27	−4.865	0.0001
c. Site = Landlocked:					
Contrast	Difference	SE	df	t.ratio	*P* value
Field.20°C–Lab.20°C	0.3	2.43	27	0.123	**0.9917**
Field.20°C–Lab.36°C	−4.5	3.21	27	−1.403	0.3534
d. Site = Mid marsh:					
Contrast	Difference	SE	df	t.ratio	*P* value
Field.20°C– Lab.20°C	−1.2	2.43	27	−0.493	**0.8752**
Field.20°C–Lab.36°C	−22.2	3.21	27	−6.923	<0.0001
April 2019:					
e. Overall model					
Effect	df	MSE	F	ges	*P* value
Site	2, 24	17.83	0.70	.025	0.506
Experiment type	1, 24	22.42	13.75	.242	0.001
Site:Experiment type	2, 24	22.42	1.30	.057	0.291
f. Field (20°C) and Lab (30°C) comparison					
Site	Difference	SE	df	t.ratio	*P* value
High marsh	2.00	2.23	24	0.896	0.3791
Landlocked	5.33	2.23	24	2.390	0.0251
Mid marsh	7.00	2.23	24	3.136	0.0045

#### Laboratory and field comparisons: April 2019

The lowest heart rates were recorded in both laboratory and field experiments conducted in April 2019 (42–49 bpm). Mussel heart rates differed significantly in the laboratory (30°C) and field (20°C) (*F*_1,24_ = 13.8, *P* = 0.001; Table 6e). The interaction between site and experiment type was not significant (*F*_2,24_ = 1.3, *P* = 0.291; Table 6e). Significant differences were observed for mussels from two sites (LHM: increase of 5.3 bpm in the laboratory, *t* = 2.4, *P* = 0.0251; MM: increase of 7.0 bpm, *t* = 3.1, *P* = 0.0045) but not for those from the third site (HM: increase of 2.0 bpm in the laboratory, *t* = 0.9, *p* = 0.3791; Fig. 6b, Table 6f). The significant differences in heart rates observed for LHM and MM mussels disappeared when data for October and April were combined (Supplementary Table S2).

#### Field comparisons in October 2018, April 2019, and September 2019

The interaction between Site and month was significant (*F* = 14.7, *P* < 0.001; Table 7a, Supplementary Fig. S4). Heart rates dropped significantly between October 2018 and April 2019 for mussels from all three sites (HM: decrease of 14.5 bpm, *t* = 7.3, *P* < 0.0001, MM: decrease of 7.5 bpm, *t* = 3.8, *P* < 0.0013, LHM: decrease of 12.3 bpm, *t* = −6.2, *P* = < 0.0001; Table 7b–d, Supplementary Fig. S4). Heart rates rose significantly in September 2019 for mussels from the HM and MM (increase of 10 bpm, *t* = 3.1, *P* < 0.0125, *df* = 29.9; 25.8, bpm, *t* = 7.7, *P* < 0.0001, *df* = 31.8, respectively) but not for those from the LHM (increase of 3.1 bpm, *t* = 1.0, *P* = 0.5688, *df* = 28.3, Kenward–Roger degrees-of-freedom method; Table 7c, Supplementary Fig. S4).

**Table 7:** (a) Two-way mixed model ANOVA with interaction between site and month of the heart rates in bpm for the ribbed mussel *G. demissa* from the HM, MM and LHM sites at Tybee Island, Georgia in Oct. 2018, April 2019 (temp, 20°C respectively), and September 2019 (temp, 40°C). *N* = 30 mussels/month, duplicate measurements/mussel for a total of 155 measurements*. Pairwise post-hoc comparisons were carried out between months for each site (b, c, and d).

a. Overall model:					
Effect	df	F	*P* value		
Site	2, 24.62	6.03	0.007		
Month	2, 26.74	52.10	<0.001		
Site:Month	4, 26.76	14.70	<0.001		
Type 3 tests, Satterthwaite's approximation					
b. Site = High marsh:					
Contrast	Difference	SE	df	t.ratio	*P* value
October–April	−14.46	1.98	51.0	−7.312	<0.0001
April – September	−10.04	3.28	29.9	−3.060	0.0125
October – September	4.42	2.81	41.5	1.571	0.2693
c. Site = Landlocked:					
Contrast	Difference	SE	df	t.ratio	*P* value
October – April	−12.33	1.98	53.5	−6.243	<0.0001
April – September	−3.14	3.07	28.3	−1.023	0.5688
October – September	9.19	2.61	35.2	3.522	0.0034
d. Site = Mid marsh:					
Contrast	Difference	SE	df	t.ratio	*P* value
October – April	−7.45	1.99	51.8	−3.752	0.0013
April– September	−25.80	3.37	31.8	−7.659	<0.0001
October– September	−18.34	2.92	43.9	−6.286	<0.0001

* Degrees-of-freedom method: Kenward–Roger.

### Part II: Variation among eight mussel aggregates in the MM

#### Mussel heart rates at laboratory temperatures of 36°C and mortality in the field

**Fig. 7:**
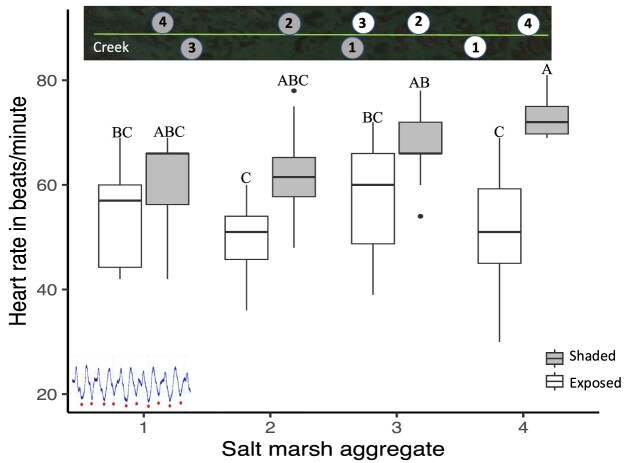
Heart rate in bpm for the mussel *G. demissa*. Box plots of heart rate in bpm for mussels from four exposed aggregates and four shaded aggregates along a 51.8 m transect line in the mid marsh off of Old Tybee Island, Georgia in 2019 and subjected to a water temperature of 36°C for an hour and a half in the laboratory. When visible, the line within the box is the median. Different letters indicate significant differences among aggregate replicates and aggregate exposure. See Fig. 5a for a description of boxplots.

Heart rates were significantly higher for mussels from shaded aggregates than for those from exposed aggregates at 36°C (66.2 ± 8.5 and 53.5 ± 10.8 bpm, respectively, *F* = 39.2, *P* < 0.0001, *n* = 80, means ± standard deviation; Table 8). A significant interaction was observed between salt marsh aggregate location along the transect line and aggregate exposure (*F* = 3.0, *P* = 0.0376; Fig. 7, Table 8). Heart rates did not differ significantly for mussels from shaded or exposed aggregates 1 through 3 but differed significantly for mussels from aggregate 4. Heart rates were significantly higher for mussels from shaded aggregate 4 than for those from exposed aggregate 4 (73.2 ± 2.9 and 50.4 ± 2.9 bpm, respectively, means ± s.e., Tukey HSD test, *P* < 0.05; Fig. 7, Table 8).

**Table 8:** Three-way ANOVA with interaction of heart rate in bpm for 80 ribbed mussels (*G. demissa*) from eight mussel aggregates (four exposed and four less exposed) located along a 51.8 m transect line in the MM at Tybee Island, GA.

Source of variation	df.	SS	MS	F	*P*
Laboratory experiment (*n* = 80) Month	1	208.013	208.013	2.5398	0.1159
Aggregate exposure	1	3213.113	3213.113	39.2321	<0.0001
Aggregate replicate	3	533.138	177.713	2.1699	0.1002
Month x Aggregate exposure	1	2.813	2.813	0.0343	0.8536
Aggregate replicate x Aggregate exposure	3	733.838	244.613	2.9867	0.0376
Month x Aggregate replicate	3	430.538	143.513	1.7523	0.1652
Month x Aggregate exposure x Aggregate replicate	3	263.138	87.713	1.0710	0.3677
Model	15	5384.588	358.973	4.38311	<0.0001
Error	64	5241.600	81.900		
Total	79	10626.188			

Mussel mortality was low among aggregates and did not vary significantly over time (*P* > 0.05; Supplementary Fig. S5). Mortality of 5% was not observed at any of the shaded aggregates but was observed at all exposed aggregates in March, August, and October 2018.

#### Mussel body temperature at the edge and center of eight mussel aggregates

**Fig. 8:**
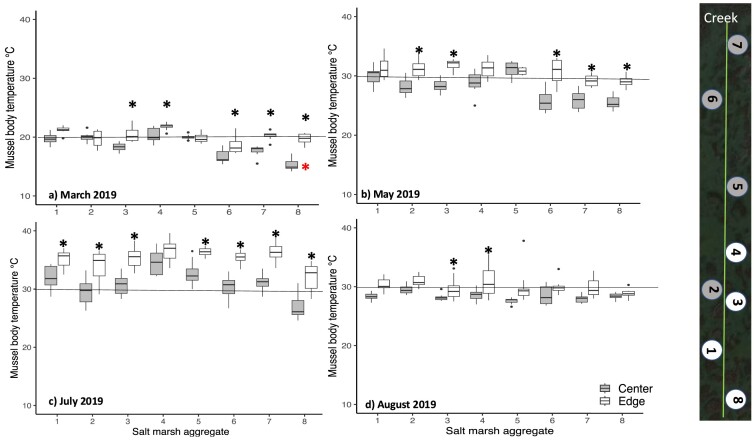
(a–d) Body temperature for the mussel *G. demissa*. Box plots of body temperature for 10 mussels from the aggregate center and 10 from the aggregate edge for each of 8 aggregates off of Old Tybee Island, Georgia collected in March, May, July and August 2019. N, the number of mussels measured per month varied from 144/month to 158/month. Asterisks indicate significant differences between the center and edge of aggregates. The 20°C and 30°C lines are lines along which mussel mortality is presumed to be non-lethal for this species. See Fig. 5a for a description of box plots.

In 2019 and 2021, with a few exceptions, mean body temperatures in the field were significantly higher for mussels at the edge of a mussel aggregate than at the center (Fig. 8a–d; Supplementary Fig. S3, Supplementary Table S3). However, there were significant interactions between the location of mussels on an aggregate (edge, center) and their location along the transect line in March 2019, May 2019, and May 2021 (*F* = 11.7, *P* = 0.0001, *F* = 3.9, *P* = 0.0007, and *F* = 4.7, *P* = 0.0373, respectively; supplementary Table S3). For instance, in March mean mussel body temperature did not differ significantly between the edges and the centers of aggregates 1, 2, and 5 but did for the centers and edges of aggregates 3, 4, 6, 7, and 8. In March, the lowest mean body temperature (15.3°C) was observed for mussels at the center of aggregate eight (Fig. 8a). There was no interaction between mussel location on an aggregate (edge, center) and their location along the transect line in July and August 2019 (*F* = 1.6, *P* = 0.1332, and *F* = 1.1, *P* = 0.3557, respectively). In both months, mean body temperatures were significantly higher for most mussels living at the aggregate edge than for those living at the aggregate center (*F* = 181.3, *P* < 0.0001, and *F* = 58.1, *P* < 0.0001, respectively, Fig. 8c and d).

The maximum recorded body temperature for an individual mussel living at the edge of an aggregate was 23°C in March, 35°C in May, 39.6°C in July and 37.8°C in August 2019, and 39°C in May 2021 (Fig. 8, Supplementary Fig. S3).

#### Hsc70, hsp70, and hsp70-3 Gene Expression in the gill tissues of ribbed mussels

**Fig. 9:**
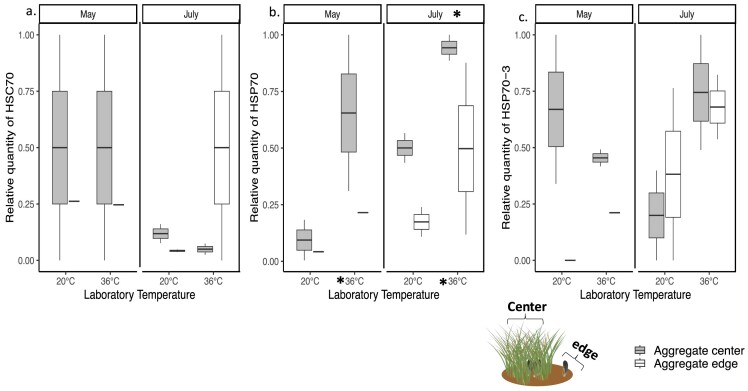
Expression of (a) hsc70 mRNA, (b) hsp70 mRNA, and (c) hsp70-3 by the mussel *G. demissa*. Box plots of the relative quantity of (a) hsc70 mRNA, (b) hsp70 mRNA, and (c) hsp70-3 mRNA gene expression at laboratory temperatures of 20°C and 36°C for gill tissue samples collected from mussels from the aggregate center and edge in May and July 2021 (total number of mussels used varied from 12 to 14). Each sample was measured in duplicate using RT-qPCR. Asterisks when present indicate significant differences between months and laboratory temperatures (*p* = 0.05). See Fig. 5a for a description of boxplots.

Despite high variation in hsc70 gene expression within a treatment, interesting trends emerged (Fig. 9). In May, hsc70 gene expression in the gills of mussels from the aggregate center was higher than for those from the aggregate edge regardless of laboratory temperature (Fig. 9a). In July 2021, hsc70 expression in mussel gill tissues dropped for all mussels regardless of their location on an aggregate except for those from the edge exposed to 36°C in the laboratory (Fig. 9a). However, these differences were not significant (Month, *F* = 1.8, *P* = 0.2210, laboratory temperature, *F* = 4.0, *P* = 0.0808, aggregate, *F* = 0.1398, *P* = 0.7182, *n* = 12, Supplementary Table S4).

Hsp70 gene expression in the mussel gills was significantly higher in July than in May (*F* = 7.5, *P* = 0.025, *n* = 12, Fig. 9b). In both May and July, hsp70 expression was significantly higher for mussels exposed to laboratory temperatures of 36°C than at 20°C regardless of their location on an aggregate (*F* = 6.4, *P* = 0.035, *n* = 12, Fig. 9b). In both months, hsp70 expression in the gill tissues was higher for mussels from the center than from the edge of an aggregate; however, these differences were not significant (*F* = 4.1, *P* = 0.0788, *n* = 12; Supplementary Table S4).

Similar trends were observed for hsp70-3 gene expression. In May, hsp70-3 expression was higher for mussels from the center of an aggregate than those from the edge regardless of temperature. In July, hsp70-3 expression was higher for mussels exposed to laboratory temperatures of 36°C than at 20°C regardless of their location on an aggregate but these differences were not significant (Month, *F* = 3.4, *P* = 0.1005, laboratory temperature, *F* = 0.3093, *P* = 0.5933, aggregate, *F* = 0.2944, *P* = 0.6022, *n* = 12; Supplementary Table S4, Fig. 9c).

## Discussion

### Environmental temperatures and mussel body temperatures

This study was interested in documenting the current environmental temperatures at three sites in a Georgia salt marsh, and determining whether ribbed mussels living in locations that differ in cordgrass height and coverage were experiencing and responding similarly to rising environmental temperatures. Results were consistent; mussels at exposed and shaded areas at the three sites were not experiencing nor responding similarly to high temperatures. As we hypothesized, the LHM site and the edges of MM mussel aggregates were the most stressful environments with the highest air temperatures (≥50°C) and the longest HTEs (2018, 58 days; 2019, 20 days, respectively) at the landlocked site. Air temperatures exceeded 45°C in the MM and HM during the summers but never reached 50°C. Furthermore, the magnitude and duration of HTEs were shorter at these latter two sites.

Despite high air temperatures in the MM, mussel body temperatures remained  ≤40°C. We acknowledge that it is difficult to tease apart the relative importance of all the factors that may be affecting mussel body temperatures in the salt marsh. However, the presence of large mussel aggregates (100–300 mussels/aggregate) beneath dense cordgrass may have played a role in keeping body temperatures low. Depending on season, body temperatures were 8–15°C cooler for mussels living in the center of large aggregates than for those at the edges. This provides additional evidence of the positive effects of living in the center of large aggregates with tall and dense cordgrass and are consistent with studies that highlight the importance of the facultative interaction between these two species (Angelini et al. 2015; Van der Heide et al. 2021, Whaley and Alldred 2023). They note that cordgrass facilitates growth of mussels by providing attachment sites and shade from the direct rays of the sun especially on hot sunny days. In turn, mussels stabilize cordgrass roots and enhance their growth. A study by Jost and Helmuth (2007) highlighted a greater role of mussel vertical body position in the salt marsh substrate over microhabitat type (vegetated or nonvegetated, well drained, or wet) in moderating body temperature. They discovered that mussels that were completely or partially buried in the substrate had the lowest body temperatures while those completely exposed had the highest body temperatures. In one instance, a completely exposed mussel was 25°C hotter than one that was completely buried. In our study, mussels at the aggregate edges may gain some benefit from being partially buried in the mud as body temperatures were at least 2–5°C cooler than air temperatures. However, the lack of shade at the edges may be a disadvantage especially during the summer months when their body temperatures approach 40°C in the middle of the day. How deep mussels are buried in the sediment may be dictated by the characteristics of the sediment (Bradley and Morris 1990). While the wet soft mud in the MM may favor partial or complete burial, the coarse sediment in the LHM may not. In addition, the presence of a few exposed mussels in an aggregate means there may be no possibility of “sharing” heat stress among mussels (see Helmuth 1998) in the LHM.

Similar results have been reported for rocky intertidal mussels living in large mussel beds. Temperatures within large mussel beds were up to 20°C lower than for those living on the surface of the bed (Jurgen and Gaylord (2016). Mislan and Wethey (2015) noted that greater contact between top layer mussels and those deeper within the bed decreases mussel body temperature on warm sunny days. They concluded that the positive effect of contact area on mussel body temperature was determined by shore level and exposure of the substratum to sunlight, with those living in the mid to low intertidal benefiting the most. Likewise, the positive interaction between cordgrass and mussels may be determined by salt marsh zone with those living in the mid and HM at high densities, partially or completely buried benefiting the most.

Windy, cloudy days, and major storms have a dampening effect on environmental temperatures. In August and September 2019 when air temperatures dropped significantly just before and during major storms, mussel body temperatures also dropped, and did not vary significantly for those living in the center and edge of 75% of the mussel aggregates. Storms may provide temporary relief from high summer temperatures. But studies are needed to examine whether increase in the frequency and intensity of storms (Leonardi et al. 2018) negates the benefits of temporary relief from heat stress in the summer.

### Temperature and mussel heart rates

Results from laboratory studies further strengthen the notion that there are multiple benefits to living in large mussel aggregates with tall and dense cordgrass for shade. Three separate laboratory experiments revealed that when laboratory temperatures were increased to 36°C, heart rates were consistently higher (72– 74 bpm) for mussels living in the HM and MM sites. This reflects an increase of 13–24 bpm above that observed for mussels at 20°C. Likewise, when temperatures rose to ≥40°C in the field, mussel heart rate increased by10–26 bpm at these two sites. Heart rates may have increased because mussels living in large aggregates with shade may rarely experience body temperatures above 32°C even during the summer months. Similar increases in heart rate with temperature have been observed for other molluscs (Somero 2012; Bjelde and Todgham 2013; Huang et al. 2015; Tagliarolo and McQuaid 2015, 2016).

Surprisingly, heart rates for mussels from the LHM remained low in the laboratory and field regardless of temperature. In one case, the heart rates of LHM mussels dropped at elevated temperature. The observed decrease in mussel heart rate may probably be due to metabolic depression (Braby and Somero 2006; Olabarria et al. 2016). Collins et al. (2020) discovered that heart rates of immersed subtidal mussels (*M. galloprovincialis*) decreased by 29% and those emersed by 50% as temperatures increased from 8 to 24°C. In contrast, heart rates of immersed or emersed intertidal mussels were unaffected by increasing temperatures from 12 to 28°C. They concluded that the subtidal and intertidal populations may have unique strategies to cope with aerial and submerged temperatures. The insensitivity to high temperatures by LHM mussels suggests that they are better adapted to warmer temperatures than MM and HM mussels.

There were some noticeable similarities among mussels from the three sites over time. In the field, mussel heart rates dropped significantly in the fall and spring when air temperatures were lower but rose again in the summer when air temperatures increased. This suggests that mussels from these sites may be responding to seasonal changes in their environment. To our knowledge, this is the first study to report seasonal differences in mussel heart rates for *G. demissa*. Interestingly, measuring the heart rate of the same mussel in the laboratory and field varied with weather conditions and site of origin. On a sunny day in October, heart rates were higher in the field than in the laboratory for mussels from the high and MM. Under similar air temperatures but on a cloudy day in April, heart rates were higher in the laboratory than in the field. These observations indicate that mussel heart rate response may depend on weather conditions in the field and should be considered when trying to compare laboratory and field studies. In contrast, regardless of weather conditions, where and when measurements were made had a negligible effect on the heart rates of mussels from the LHM site.

### Temperature and Hsc70, hsp70, and hsp70-3 gene expression

Although current data are limited, to our knowledge this is the first study that observed interesting trends in hsc70, hsp70, and hsp70-3 expression in *G. demissa* based on temperature, location on an aggregate, and month. At 36°C in the laboratory, hsc gene expression in the gills was higher for mussels found less than a few meters apart on the center and edge of an aggregate. Hsc70 gene expression was also higher in May than in July. Similar high hsc70 expression in tissues of other molluscs have been observed (Franzellitti and Fabbri 2005, Liu and Chen 2013). Likewise, laboratory temperatures of 36°C led to high hsp70 and hsp70-3 gene expression in mussel gill tissues. In July, the magnitude of the increase in hsp70 was significantly higher for mussels from the center of an aggregate; once again suggesting that they may rarely experience environmental temperatures above 32°C in their microhabitat.

Differences in gene expression were more pronounced in the summer regardless of location on an aggregate. Past studies on *G.demissa* (Fields and Eraso 2020), and other molluscs, *M. californianus* (Hoffman and Somero 1995), *M. Trossulus* (Buckley et al. 2001), *M. galloprovincialis* (Franzellitti and Fabbri 2005) and *Crassostrea gigas* (Hamdoun et al. 2003) also noted significantly higher levels of Hsp70 in the summer than in the winter or spring. They concluded that these molluscs may have an elaborate molecular strategy where inducible Hsp70 genes are expressed at low levels during the spring and their synthesis increases during the summer months as temperatures rise.

### Limitations, future studies, and conclusions

Counts of mussels in large aggregates may have been underestimated as it was impossible to count those that were completely buried in the middle of aggregates. Heart rates were generally made using the same individuals in the laboratory and the field, excluding September when heart rates where only measured in the field. Though results suggest that the effects on this study may have been minimal, in the future, field studies should include heart rate measurements from a fresh set of mussels. Thus, the possibility of legacy effects can be addressed. The logistics of carrying out field experiments can be difficult; however, we suggest that while recording mussel heart rates in the laboratory and field, air, water, and body temperatures should be measured continuously and concurrently (Jost and Helmuth 2007; Collins et al. 2020). The ramping rates used in the current study were acute and may be only an approximation of what *G.demissa* may experience in nature. We thus suggest that future studies should use ramping rates of 4, 6, and 9°C/hr. that reflect observed ramping rates at the 3 sites. Finally, studies with much larger sample sizes are needed to confirm the results obtained for hsc70, hsp70, and hsp70-3 gene expression in the gills of *G.demissa* exposed to high temperatures in the laboratory and field.

In conclusion, significant increases in body temperatures, heart rates, and hsp70 and hsp70-3 gene expression were observed for mussels exposed to elevated temperatures in the laboratory and field for two of the three sites. Mussel heart rates also varied seasonally and with weather conditions. The presence of large mussel aggregates beneath tall and dense cordgrass for shade appeared to mitigate the effects of extremely high air temperatures that coincide with low tides in the middle of the day, especially during the summer months. Mussels living in the center of large aggregates in the MM thus have the best chance of survival, while those living at the edges may have the worst chance of survival as temperatures continue to rise. That is if the interaction between the two species in the MM is not destabilized by the combined effects of rising temperatures and coastal development. Mussels at the LHM site may not be as sensitive to temperature changes or unpredictable weather conditions as those from the other two sites, because the combined effects of low cordgrass density and poor recruitment may have destabilized this system. Given the value of salt marsh ecosystems (Gedan et al. 2009; Erwin 2009; Barbier et al. 2011; Temmerman et al. 2023), and the significant role of the ribbed mussel *G. demissa* in maintaining species diversity (Kreeger et al. 2015; Angelini et al. 2016; Williams et al. 2023), loss of cordgrass (O'Donnell and Schalles 2016) and the accompanying decrease in mussel aggregate size could lower overall mussel survival along the US Atlantic and Gulf coast.

## Supplementary Material

obae031_Supplemental_Files
