# Supplementary material for: The Physiological and Biochemical Response of Ribbed Mussels to Rising Temperatures: Benefits of Salt Marsh Cordgrass
Source: Integr Org Biol. 2024 Aug 21;6(1):obae031. doi: 10.1093/iob/obae031 (PMC11398905; doi:10.1093/iob/obae031)
Supplement: obae031_Supplemental_Files [file obae031_supplemental_files.zip › Supplementary Figure legends.docx]

Supplementary Figures

Supplemental Figure 1: Landlocked site showing roads on all sides.

Supplemental Figure 2: Set-up for mussel heart rate measurements.

Supplemental Figure 3: Mussel body temperature at the edge and center of two salt marsh aggregates in the mid marsh, May 2021.

Supplemental Figure 4: Field measurements: Violin plots of heart rate in beats per minute for ribbed mussels, *Geukensia demissa* at the LHM, MM, and HM sites off of old Tybee Road, Tybee Island, Georgia in October 2018, April and September 2019. Air temperatures in the field at the time of the experiment were 20, 20, and 40°C respectively. Asterisks indicate significant differences among locations and month.

Supplemental Figure 5: Box plots of mussel mortality (%) for the ribbed mussel (*Geukensia demissa*) from shaded and exposed sites in the mid marsh at Tybee Island, Georgia in March, April, August and September 2018.
