# Supplementary material for: The Physiological and Biochemical Response of Ribbed Mussels to Rising Temperatures: Benefits of Salt Marsh Cordgrass
Source: Integr Org Biol. 2024 Aug 21;6(1):obae031. doi: 10.1093/iob/obae031 (PMC11398905; doi:10.1093/iob/obae031)
Supplement: obae031_Supplemental_Files [file obae031_supplemental_files.zip › Supplementary figures.pptx]

## Slide 1
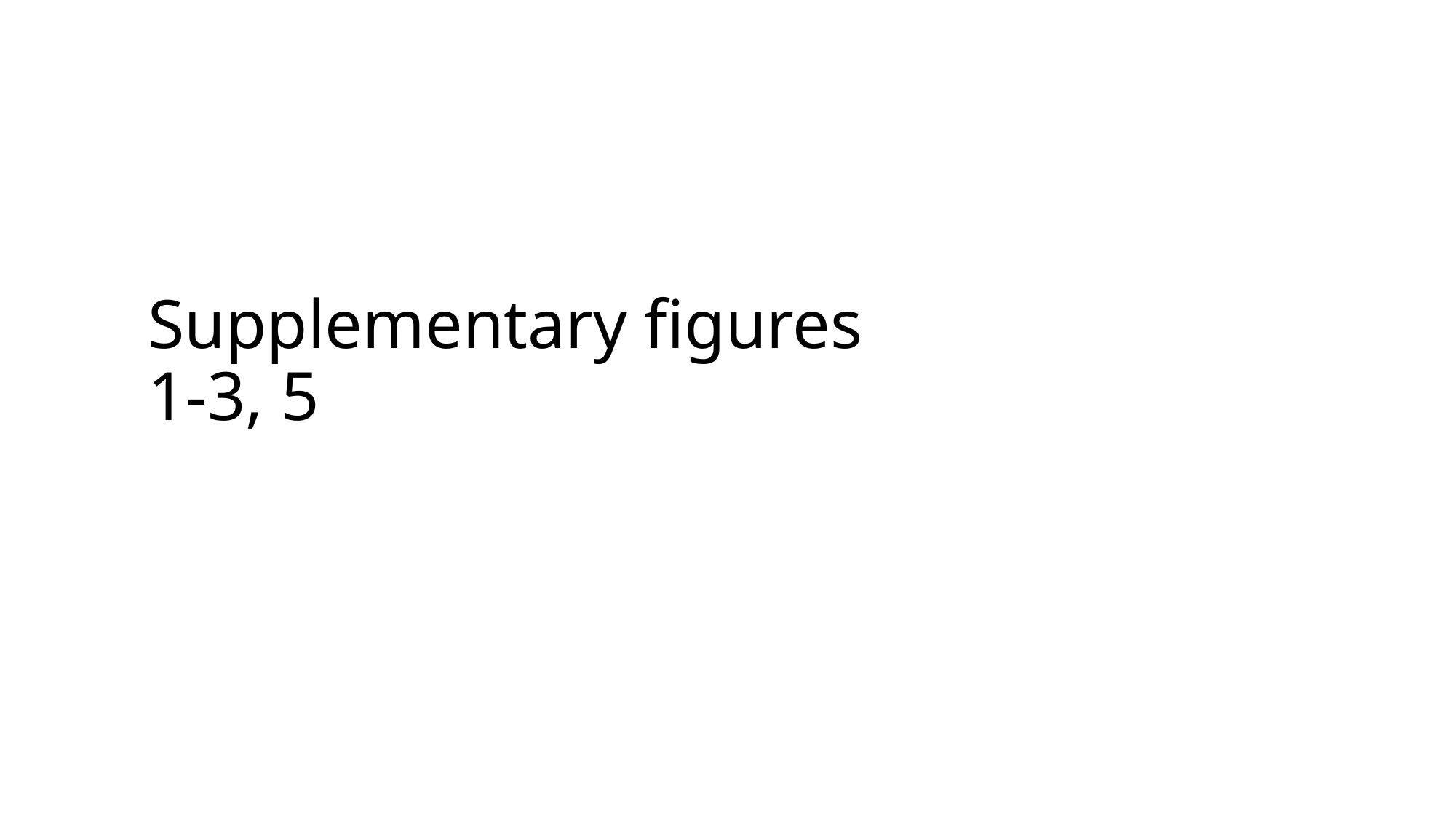

# Supplementary figures 1-3, 5

## Slide 2
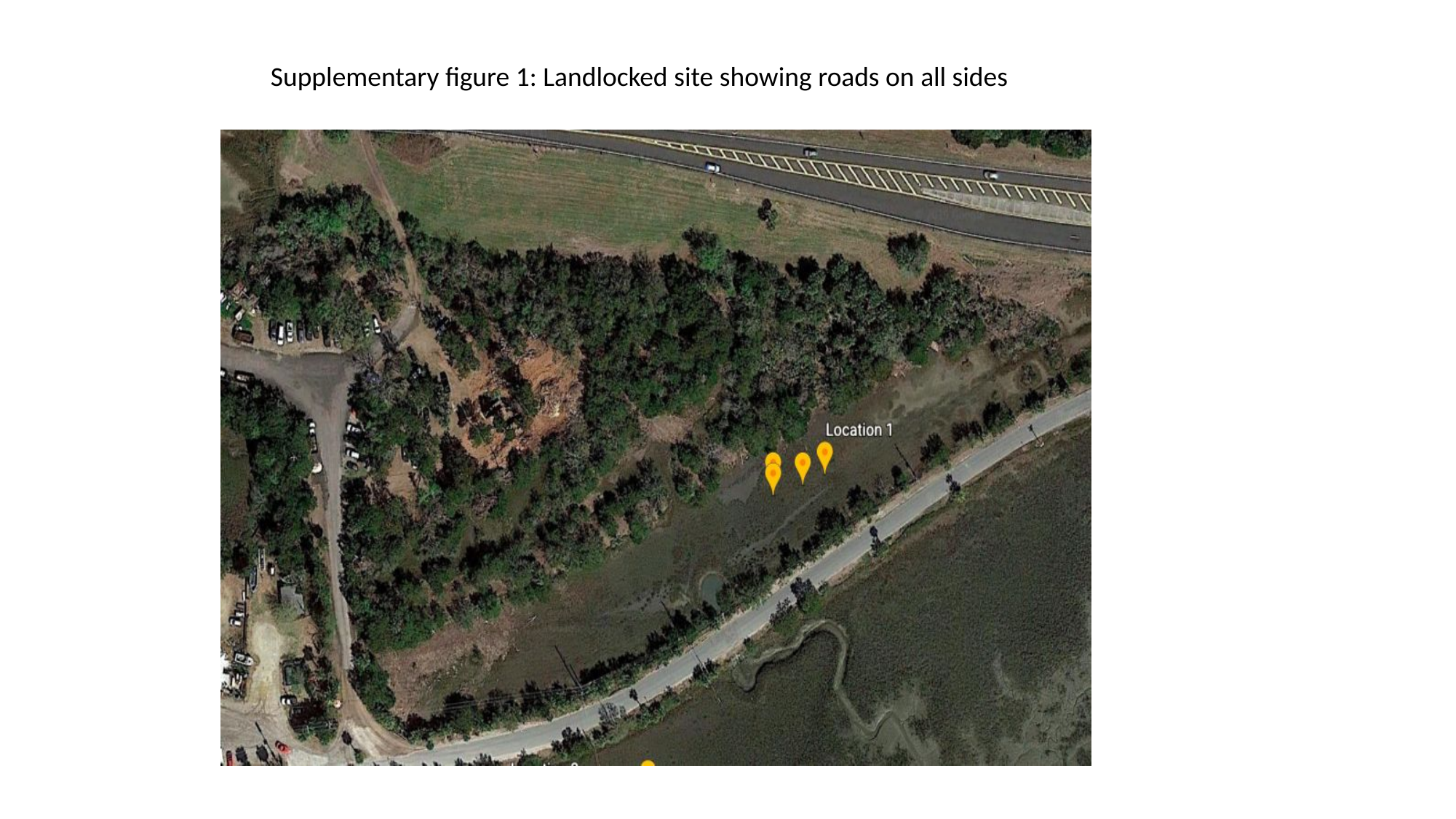

Supplementary figure 1: Landlocked site showing roads on all sides

## Slide 3
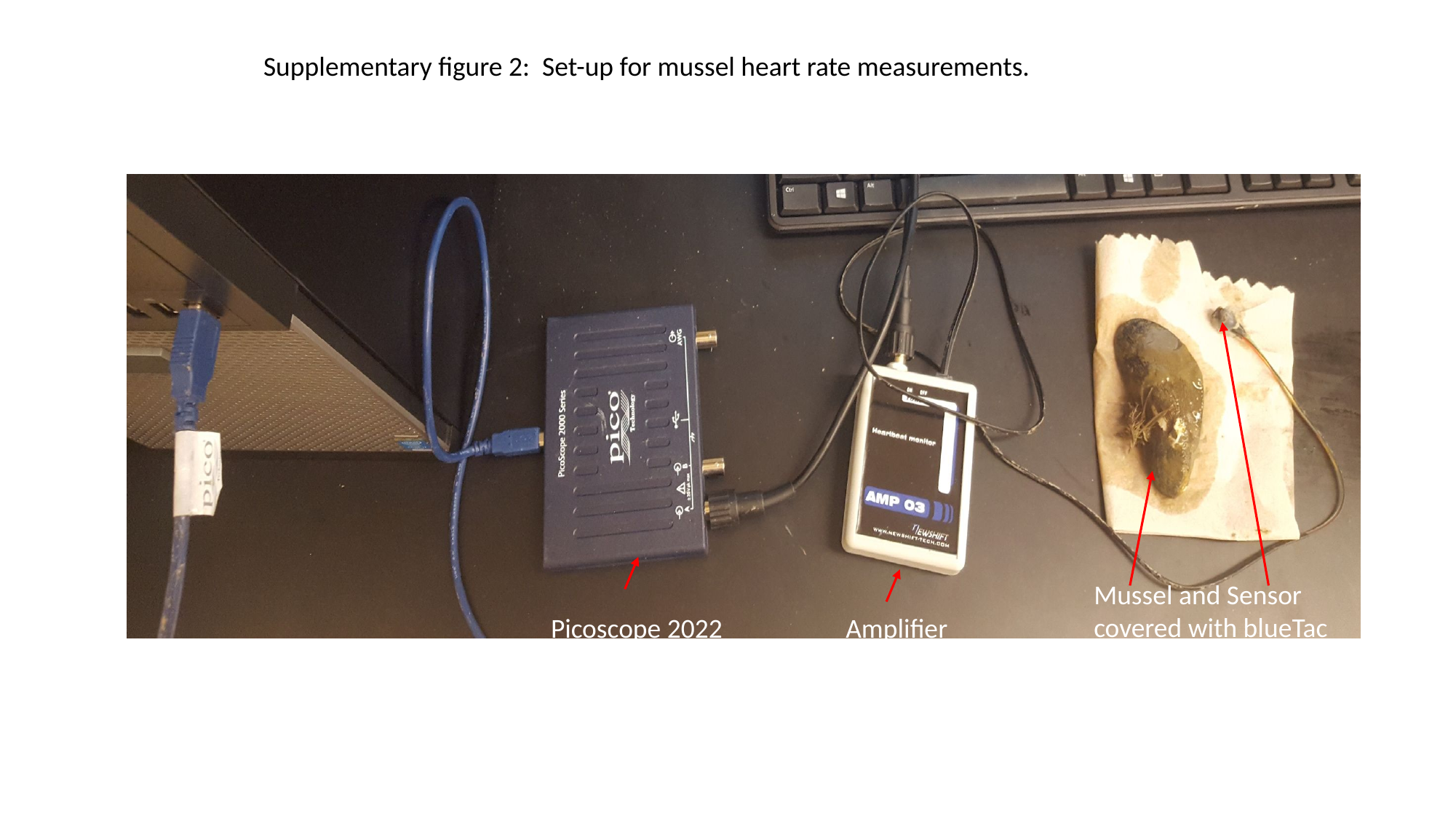

Supplementary figure 2: Set-up for mussel heart rate measurements.
Mussel and Sensor covered with blueTac
Picoscope 2022
Amplifier

## Slide 4
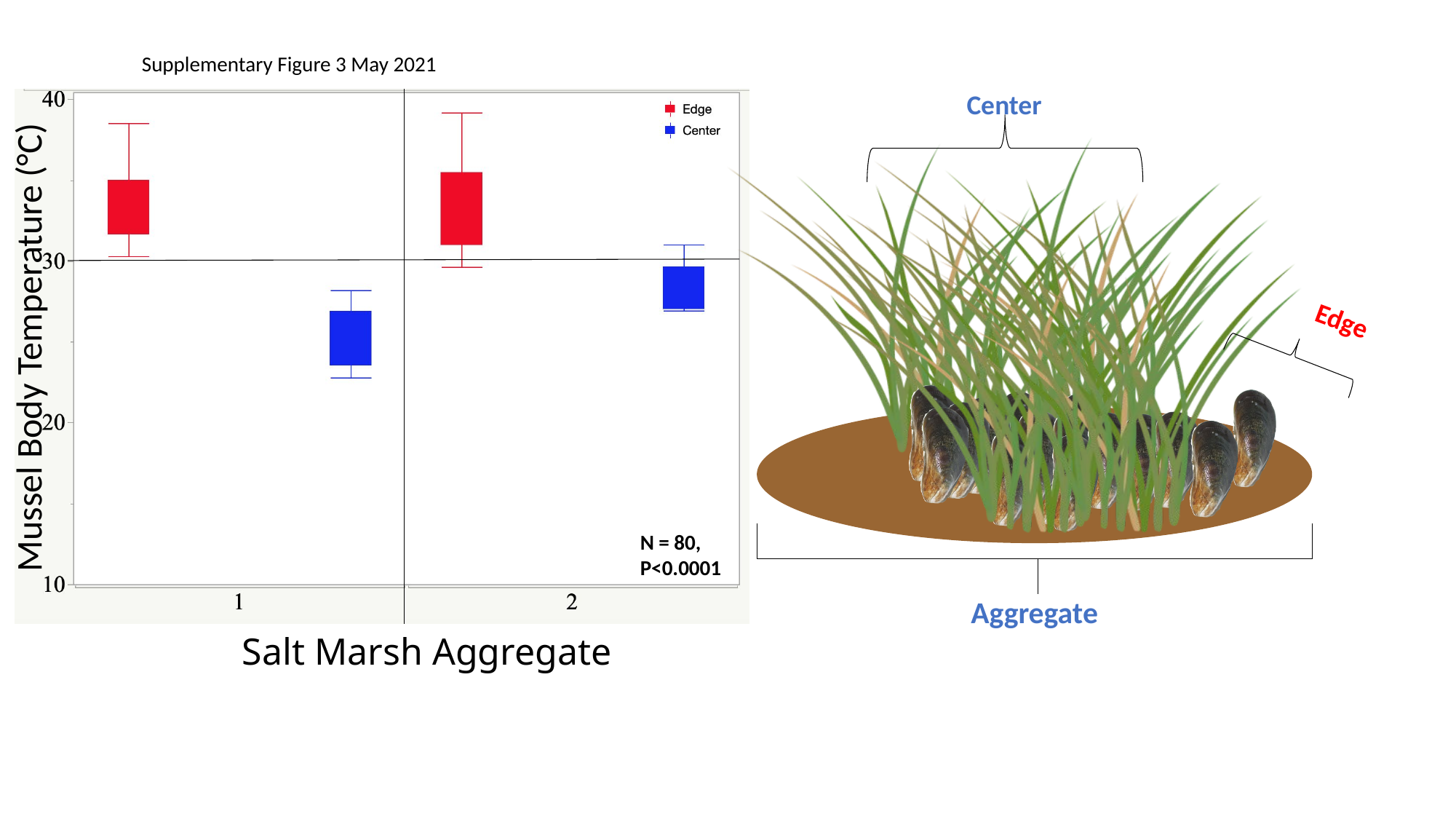

Supplementary Figure 3 May 2021
 Center
Edge
Aggregate
Mussel Body Temperature (°C)
N = 80,
P<0.0001
Salt Marsh Aggregate

## Slide 5
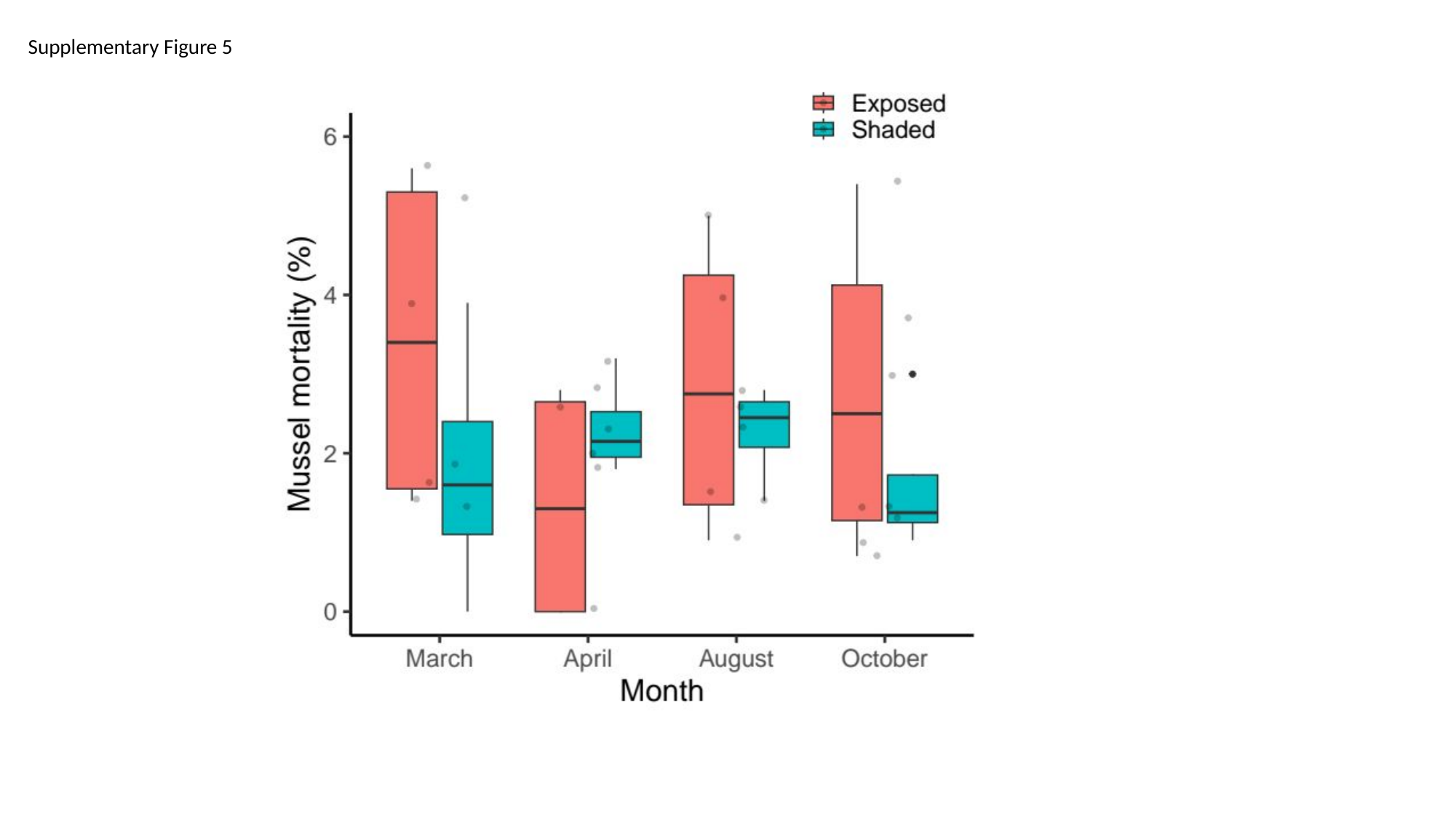

Supplementary Figure 5
