# Supplementary material for: The Physiological and Biochemical Response of Ribbed Mussels to Rising Temperatures: Benefits of Salt Marsh Cordgrass
Source: Integr Org Biol. 2024 Aug 21;6(1):obae031. doi: 10.1093/iob/obae031 (PMC11398905; doi:10.1093/iob/obae031)
Supplement: obae031_Supplemental_Files [file obae031_supplemental_files.zip › Supplementary Tables.docx]

**Supplementary Tables**

**Supplementary Table 1.**  Coordinates of 1m x 1m plots in the landlocked, high marsh, and mid marsh locations at Tybee Island, Georgia.

| Coordinates of Plots (1m x 1m) | | | | | | | | |
| --- | --- | --- | --- | --- | --- | --- | --- | --- |
| Location | Plot 1 | | Plot 2 | | Plot 3 | | Plot 4 | |
|  | Latitude | Longitude | Latitude | Longitude | Latitude | Longitude | Latitude | Longitude |
| Landlocked | 32.01475 | -80.880117 | 32.014733 | -80.8802 | 32.014717 | -80.8803 | 32.014683 | -80.880317 |
| High marsh | 32.013867 | -80.880967 | 32.013883 | -80.88085 | 32.0139 | -80.880783 | 32.013917 | -80.880783 |
| Mid marsh | 32.013767 | -80.880733 | 32.013733 | -80.88075 | 32.0137 | -80.880783 | 32.013667 | -80.88085 |

Supplementary Table 2: Combined three-way repeated measures ANOVA for October 2018 and April 2019

lab-field heart rate data for the mussel *Geukensia demissa*

Overall Model:

| Effect | df | MSE | F | ges | p.value |
| --- | --- | --- | --- | --- | --- |
| Month | 1, 50 | 38.82 | 192.62 | 0.660 | <.001 |
| Site | 2, 50 | 38.82 | 2.54 | 0.049 | .089 |
| Month:Site | 2, 50 | 38.82 | 5.77 | 0.104 | .006 |
| Temperature | 1, 50 | 38.33 | 66.00 | 0.396 | <.001 |
| Month:Temperature | 1, 50 | 38.33 | 16.36 | 0.140 | <.001 |
| Site:Temperature | 2, 50 | 38.33 | 5.99 | 0.106 | .005 |
| Month:Site:Temperature | 2, 50 | 38.33 | 4.93 | 0.089 | .011 |

ges = generalized eta squared

Month = October 2018

| Contrast | Difference | SE | df | t.ratio | p.value |
| --- | --- | --- | --- | --- | --- |
| High marsh Low.Field - Landlocked Low.Field | 2.40 | 1.98 | 50 | 1.212 | 0.8289 |
| High marsh Low.Field - Mid marsh Low.Field | 8.80 | 2.03 | 50 | 4.326 | 0.0010 |
| High marsh Low.Field - High marsh High.Lab | -15.60 | 2.77 | 50 | -5.634 | <.0001 |
| High marsh Low.Field - Landlocked High.Lab | -2.10 | 2.78 | 50 | -0.756 | 0.9735 |
| High marsh Low.Field - Mid marsh High.Lab | -13.87 | 2.89 | 50 | -4.798 | 0.0002 |
| Landlocked Low.Field - Mid marsh Low.Field | 6.40 | 2.03 | 50 | 3.146 | 0.0314 |
| Landlocked Low.Field - High marsh High.Lab | -18.00 | 2.78 | 50 | -6.481 | <.0001 |
| Landlocked Low.Field - Landlocked High.Lab | -4.50 | 2.77 | 50 | -1.625 | 0.5862 |
| Landlocked Low.Field - Mid marsh High.Lab | -16.27 | 2.89 | 50 | -5.628 | <.0001 |
| Mid marsh Low.Field - High marsh High.Lab | -24.40 | 2.82 | 50 | -8.663 | <.0001 |
| Mid marsh Low.Field - Landlocked High.Lab | -10.90 | 2.82 | 50 | -3.870 | 0.0041 |
| Mid marsh Low.Field - Mid marsh High.Lab | -22.67 | 2.92 | 50 | -7.767 | <.0001 |
| High marsh High.Lab - Landlocked High.Lab | 13.50 | 3.39 | 50 | 3.979 | 0.0029 |
| High marsh High.Lab - Mid marsh High.Lab | 1.73 | 3.49 | 50 | 0.497 | 0.9961 |
| Landlocked High.Lab - Mid marsh High.Lab | -11.77 | 3.49 | 50 | -3.376 | 0.0170 |

Month = April 2019:

| Contrast | Difference | SE | df | t.ratio | p.value |
| --- | --- | --- | --- | --- | --- |
| High marsh Low.Field - Landlocked Low.Field | 0.00 | 2.09 | 50 | 0.000 | 1.0000 |
| High marsh Low.Field - Mid marsh Low.Field | 1.67 | 2.09 | 50 | 0.799 | 0.9665 |
| High marsh Low.Field - High marsh High.Lab | -2.00 | 2.92 | 50 | -0.685 | 0.9828 |
| High marsh Low.Field - Landlocked High.Lab | -5.33 | 2.93 | 50 | -1.822 | 0.4616 |
| High marsh Low.Field - Mid marsh High.Lab | -5.33 | 2.93 | 50 | -1.822 | 0.4616 |
| Landlocked Low.Field - Mid marsh Low.Field | 1.67 | 2.09 | 50 | 0.799 | 0.9665 |
| Landlocked Low.Field - High marsh High.Lab | -2.00 | 2.93 | 50 | -0.683 | 0.9830 |
| Landlocked Low.Field - Landlocked High.Lab | -5.33 | 2.92 | 50 | -1.827 | 0.4581 |
| Landlocked Low.Field - Mid marsh High.Lab | -5.33 | 2.93 | 50 | -1.822 | 0.4616 |
| Mid marsh Low.Field - High marsh High.Lab | -3.67 | 2.93 | 50 | -1.252 | 0.8088 |
| Mid marsh Low.Field - Landlocked High.Lab | -7.00 | 2.93 | 50 | -2.391 | 0.1791 |
| Mid marsh Low.Field - Mid marsh High.Lab | -7.00 | 2.92 | 50 | -2.399 | 0.1765 |
| High marsh High.Lab - Landlocked High.Lab | -3.33 | 3.58 | 50 | -0.932 | 0.9362 |
| High marsh High.Lab - Mid marsh High.Lab | -3.33 | 3.58 | 50 | -0.932 | 0.9362 |
| Landlocked High.Lab - Mid marsh High.Lab | 0.00 | 3.58 | 50 | 0.000 | 1.0000 |

Tukey method for comparison, Low and high refers to temperature.

Supplementary Table 3: Two-way ANOVA with interaction of the body temperatures of the ribbed mussel

*Geukensia demissa* living at the edge and center of mussel aggregates in the mid marsh at Tybee Island,

Georgia. Measurements were made in the field on mussels from eight aggregates from March through August

2019 and from two mussel aggregates in May 2021.

| Source of variation | df | SS | MS | F | *P* |
| --- | --- | --- | --- | --- | --- |
| **Mar-19** |  |  |  |  |  |
| Mussel location on aggregate (edge or center) | 1 | 102.299 | 102.299 | 118.2 | <0.0001 |
| Aggregate | 7 | 225.564 | 32.223 | 37.2 | <0.0001 |
| Mussel location on aggregate x Aggregate | 7 | 71.136 | 10.162 | 11.7 | <0.0001 |
| Model | 15 | 418.912 | 27.926 | 32.3 | <0.0001 |
| Error | 138 | 119.392 | 0.865 |  |  |
| Total | 153 | 538.304 |  |  |  |
|  |  |  |  |  |  |
| **May-19** |  |  |  |  |  |
| Mussel location on aggregate (edge or center) | 1 | 242.091 | 242.091 | 118.7 | <0.0001 |
| Aggregate | 7 | 238.656 | 34.094 | 16.7 | <0.0001 |
| Mussel location on aggregate x Aggregate | 7 | 55.306 | 7.900 | 3.9 | 0.0007 |
| Model | 15 | 614.969 | 40.998 | 20.1 | <0.0001 |
| Error | 128 | 260.967 | 2.039 |  |  |
| Total | 143 | 875.936 |  |  |  |
|  |  |  |  |  |  |
| **Jul-19** |  |  |  |  |  |
| Mussel location on aggregate (edge or center) | 1 | 659.826 | 659.826 | 181.3 | <0.0001 |
| Aggregate | 7 | 486.203 | 69.458 | 19.1 | <0.0001 |
| Mussel location on aggregate x Aggregate | 7 | 41.387 | 5.912 | 1.6 | 0.1332 |
| Model | 15 | 1190.165 | 79.344 | 21.8 | <0.0001 |
| Error | 138 | 502.111 | 3.639 |  |  |
| Total | 153 | 1692.276 |  |  |  |
|  |  |  |  |  |  |
| **Aug-19** |  |  |  |  |  |
| Mussel location on aggregate (edge or center) | 1 | 110.805 | 110.805 | 58.1 | <0.0001 |
| Aggregate | 7 | 40.979 | 5.854 | 3.1 | 0.0049 |
| Mussel location on aggregate x Aggregate | 7 | 14.914 | 2.131 | 1.1 | 0.3557 |
| Model | 15 | 167.133 | 11.142 | 5.8 | <0.0001 |
| Error | 142 | 270.838 |  |  |  |
| Total | 157 | 437.971 |  |  |  |
|  |  |  |  |  |  |
| **May-21** |  |  |  |  |  |
| Mussel location on aggregate (edge or center) | 1 | 454.276 | 454.276 | 87.3 | <0.0001 |
| Aggregate | 1 | 15.876 | 15.876 | 3.0 | 0.0893 |
| Mussel location on aggregate x Aggregate | 1 | 24.336 | 24.336 | 4.7 | 0.0373 |
| Model | 3 | 494.488 | 164.829 | 31.7 | <0.0001 |
| Error | 36 | 187.416 | 5.206 |  |  |
| Total | 39 | 681.904 |  |  |  |
|  |  |  |  |  |  |

Supplementary Table 4. Three-way ANOVA for hsp70, hsp70-3, and hsc70 expressed

in the gills of the mussel *Geukensia demissa* collected from Tybee Island, Georgia, in

October 2018 and April 2019.

Hsc70

| Effect | df | SS | MS | F ratio | P value |
| --- | --- | --- | --- | --- | --- |
| Temperature (20, 36°C) | 1 | 0.0460 | 0.0460 | 0.29 | 0.6022 |
| Exposure (Center, Edge) | 1 | 0.0484 | 0.0484 | 0.31 | 0.5933 |
| Month (May, July) | 1 | 0.5387 | 0.5387 | 3.44 | 0.1005 |
| Model | 3 | 0.6331 | 0.2110 | 1.35 | 0.3255 |
| Error | 8 | 1.2510 | 0.1564 |  |  |
| Total | 11 | 1.8841 |  |  |  |

Hsp70

| Effect | df | SS | MS | F ratio | P value |
| --- | --- | --- | --- | --- | --- |
| Temperature (20, 36°C) | 1 | 0.3366 | 0.3366 | 6.38 | 0.0354 |
| Exposure (Center, Edge) | 1 | 0.2137 | 0.2137 | 4.06 | 0.0788 |
| Month (May, July) | 1 | 0.3971 | 0.3971 | 7.53 | 0.0252 |
| Model | 3 | 0.9474 | 0.3158 | 5.99 | 0.0192 |
| Error | 8 | 0.4216 | 0.0527 |  |  |
| Total | 11 | 1.3690 |  |  |  |

Hsp70-3

| Effect | df | SS | MS | F ratio | P value |
| --- | --- | --- | --- | --- | --- |
| Temperature (20, 36°C) | 1 | 0.0122 | 0.0122 | 0.14 | 0.7182 |
| Exposure (Center, Edge) | 1 | 0.3504 | 0.3504 | 3.99 | 0.0808 |
| Month (May, July) | 1 | 0.1548 | 0.1548 | 1.76 | 0.2210 |
| Model | 3 | 0.5175 | 0.1725 | 1.94 | 0.1981 |
| Error | 8 | 0.7026 | 0.0878 |  |  |
| Total | 11 | 1.2202 |  |  |  |
