# Supplementary figures and images for: The Physiological and Biochemical Response of Ribbed Mussels to Rising Temperatures: Benefits of Salt Marsh Cordgrass
Source: Integr Org Biol. 2024 Aug 21;6(1):obae031. doi: 10.1093/iob/obae031 (PMC11398905; doi:10.1093/iob/obae031)

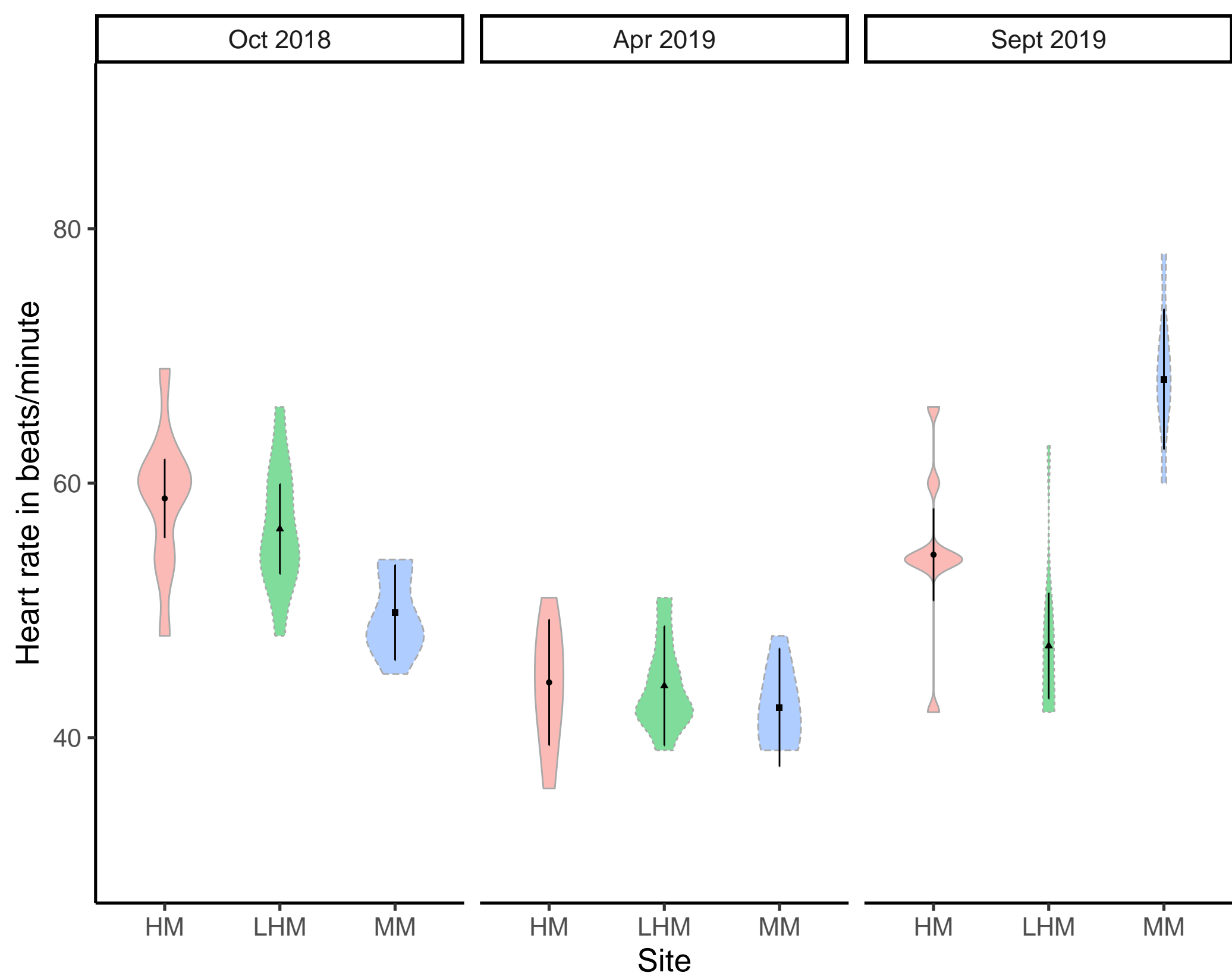

Supplement: obae031_Supplemental_Files [file obae031_supplemental_files.zip › Supplementary Figure 4.pdf]
